# Microtubule-associated septin complexes modulate kinesin and dynein motility with differential specificities

**DOI:** 10.1016/j.jbc.2023.105084

**Published:** 2023-07-24

**Authors:** Yani Suber, Md Noor A. Alam, Konstantinos Nakos, Priyanka Bhakt, Elias T. Spiliotis

**Affiliations:** Department of Biology, Drexel University, Philadelphia, Pennsylvania, USA

**Keywords:** septins, microtubules, dynein, kinesin, microtubule-associated proteins, membrane traffic, neurons, Golgi

## Abstract

Long-range membrane traffic is guided by microtubule-associated proteins and posttranslational modifications, which collectively comprise a traffic code. The regulatory principles of this code and how it orchestrates the motility of kinesin and dynein motors are largely unknown. Septins are a large family of GTP-binding proteins, which assemble into complexes that associate with microtubules. Using single-molecule *in vitro* motility assays, we tested how the microtubule-associated SEPT2/6/7, SEPT2/6/7/9, and SEPT5/7/11 complexes affect the motilities of the constitutively active kinesins KIF5C and KIF1A and the dynein-dynactin-bicaudal D (DDB) motor complex. We found that microtubule-associated SEPT2/6/7 is a potent inhibitor of DDB and KIF5C, preventing mainly their association with microtubules. SEPT2/6/7 also inhibits KIF1A by obstructing stepping along microtubules. On SEPT2/6/7/9-coated microtubules, KIF1A inhibition is dampened by SEPT9, which alone enhances KIF1A, showing that individual septin subunits determine the regulatory properties of septin complexes. Strikingly, SEPT5/7/11 differs from SEPT2/6/7, in permitting the motility of KIF1A and immobilizing DDB to the microtubule lattice. In hippocampal neurons, filamentous SEPT5 colocalizes with somatodendritic microtubules that underlie Golgi membranes and lack SEPT6. Depletion of SEPT5 disrupts Golgi morphology and polarization of Golgi ribbons into the shaft of somato-proximal dendrites, which is consistent with the tethering of DDB to microtubules by SEPT5/7/11. Collectively, these results suggest that microtubule-associated complexes have differential specificities in the regulation of the motility and positioning of microtubule motors. We posit that septins are an integral part of the microtubule-based code that spatially controls membrane traffic.

Long-range transport of membrane vesicles and organelles takes place on the microtubule cytoskeleton, a meshwork of α-/β-tubulin polymers, and is mediated by the kinesin and dynein motors (1, 2). Spatial navigation of the microtubule network is critical for the accurate and timely delivery of cargo, but how motor movement is spatially controlled on microtubules is poorly understood. Growing evidence indicates that microtubules are a heterogeneous network, a mosaic of polymers with varying compositions of tubulin isotypes, posttranslational modifications, and microtubule-associated proteins (MAPs) (3, 4, 5). Collectively, this biochemical diversity provides a spatial code, which directs membrane traffic by determining the microtubule tracks and motile properties of motors and their cargo (6, 7, 8, 9).

*In vitro* reconstitution of kinesin and dynein motility has begun to provide key insights into the mechanisms by which the binding and movement of motors is regulated on microtubules. Recent work has revealed that MAPs selectively and differentially regulate the motility of kinesin motors (5, 7, 10, 11). MAP7/ensconsin promotes the recruitment and motility of kinesin-1, which is inhibited by most other MAPs (12, 13, 14, 15, 16, 17, 18). Similarly, the motility of the kinesin-3 motor KIF1A is restricted or permitted by different MAPs (13, 19, 20, 21). Dynein–dynactin motor complexes are also differentially affected by MAPs (12, 13, 14, 22, 23, 24, 25). These findings have raised the importance of a comprehensive understanding of the selective regulation of motors by MAPs, which guides the movement and positioning of membrane cargo in the microtubule network.

Septins are GTP-binding proteins, which assemble into multimeric nonpolar oligomers and filaments that associate with microtubules and actin filaments (26, 27, 28). Mammalian septins comprise a large family of 13 paralogous genes, which are categorized into four distinct groups based on sequence similarity (29). Septin complexes consist of subunits from each of the four groups, which can assemble minimally into hetero-hexamers or hetero-octamers, head-to-head dimers of hetero-trimers or hetero-tetramers (30, 31, 32, 33). Subunits of the same septin group can replace one another within their corresponding positions, generating a diversity of complexes (34, 35). Alternative complexes with multiple subunits from the same group have been reported and may arise from disproportionate expression of different septins in certain cell types (36). The evolutionarily expansion of septin genes and isoforms suggests that septin subunits bestow distinct properties and functions upon their respective complexes (37, 38, 39, 40). However, the functional specificity of distinct septin subunits and complexes is little understood and explored in mammalian cells.

Mammalian septins associate with subsets of microtubules regulating their spatial organization and dynamics (41). In hippocampal neurons, septin 9 (SEPT9) localizes to dendritic microtubules and reinforces the polarity of membrane traffic by impeding the transport of axonal cargo of kinesin-1 (KIF5C) and promoting the movement of dendritic cargo of kinesin-3 (KIF1A) (19, 42). *In vitro* motility assays revealed that microtubule-associated SEPT9 differentially regulates kinesin motility, inhibiting KIF5C, enhancing KIF1A, and having no impact on kinesin-2 KIF17 (19). Additionally, SEPT9 reduced the velocity and run length of the activated dynein-dynactin-bicaudal D (DDB) motor complex (19). Unexpectedly, these findings showed that a microtubule-associated septin can differentially modulate the motility of microtubule motors, raising the questions of whether SEPT9 functions similarly in heteromeric septin complexes and whether different septin complexes have distinct regulatory properties. Investigating these questions may not only shed light on the functional specificity of septin complexes and subunits but also reveal a septin-based code for the spatial control of membrane traffic (43).

Here, we sought to examine how three microtubule-associated septin complexes with distinct subunit compositions affect the motility of kinesin and dynein motors. Using *in vitro* reconstitution assays of motor motility, we compared the microtubule-associated SEPT2/6/7 and SEPT5/7/11 with one another, and we also asked how septin subunits with opposing effects on KIF1A motility function when they are part of the same complex. We found that SEPT5/7/11 differs from SEPT2/6/7 in being largely permissive to KIF1A motility and promoting microtubule tethering of dynein and kinesin motors. We also report that in SEPT2/6/7/9 complexes, the SEPT9 and SEPT2/6/7 subunits counter each other in enhancing and inhibiting KIF1A motility, which results in a blended effect. In agreement with the distinct properties of SEPT5/7/11 complexes, SEPT5 localizes to a subset of neuronal microtubules that lack SEPT6 and promotes Golgi ribbon morphology and localization in the somatodendritic compartment of hippocampal neurons. Collectively, our results reveal that microtubule-associated septins have differential specificities in modulating kinesin- and dynein-driven motility, which are determined by the combinatorial identity of their subunits.

## Results

### Sept2/6/7 inhibits the motility of kinesins Kif5C and Kif1A and DDB

We sought to examine whether heteromeric microtubule-associated septin complexes can modulate the motility of microtubule motors using *in vitro* motility assays and total internal reflection fluorescence (TIRF) microscopy. The SEPT2/6/7 complex is a well-characterized complex, which assembles into higher order multimers and interacts with microtubules (44). We purified recombinant mCherry-tagged SEPT2/6/7 complexes (Fig. S1*A*), which coated taxol-stabilized microtubules in a concentration-dependent manner (Fig. S1, *D* and *E*). Motility assays were performed after decoration of microtubules with 100 nM mCherry-SEPT2/6/7, which resulted in uniform microtubule coating that persisted in the buffer conditions of the motility assay (Fig. S1, *D* and *E*); SEPT2/6/7 concentration was at the low end of the intracellular range (∼200–800 nM) of septin concentrations (45, 46). In the microtubule-bound mCherry-SEPT2/6/7 complexes, presence of SEPT6 and SEPT7 was verified by immunolabeling (Fig. S1*F*).

**Figure 1:**
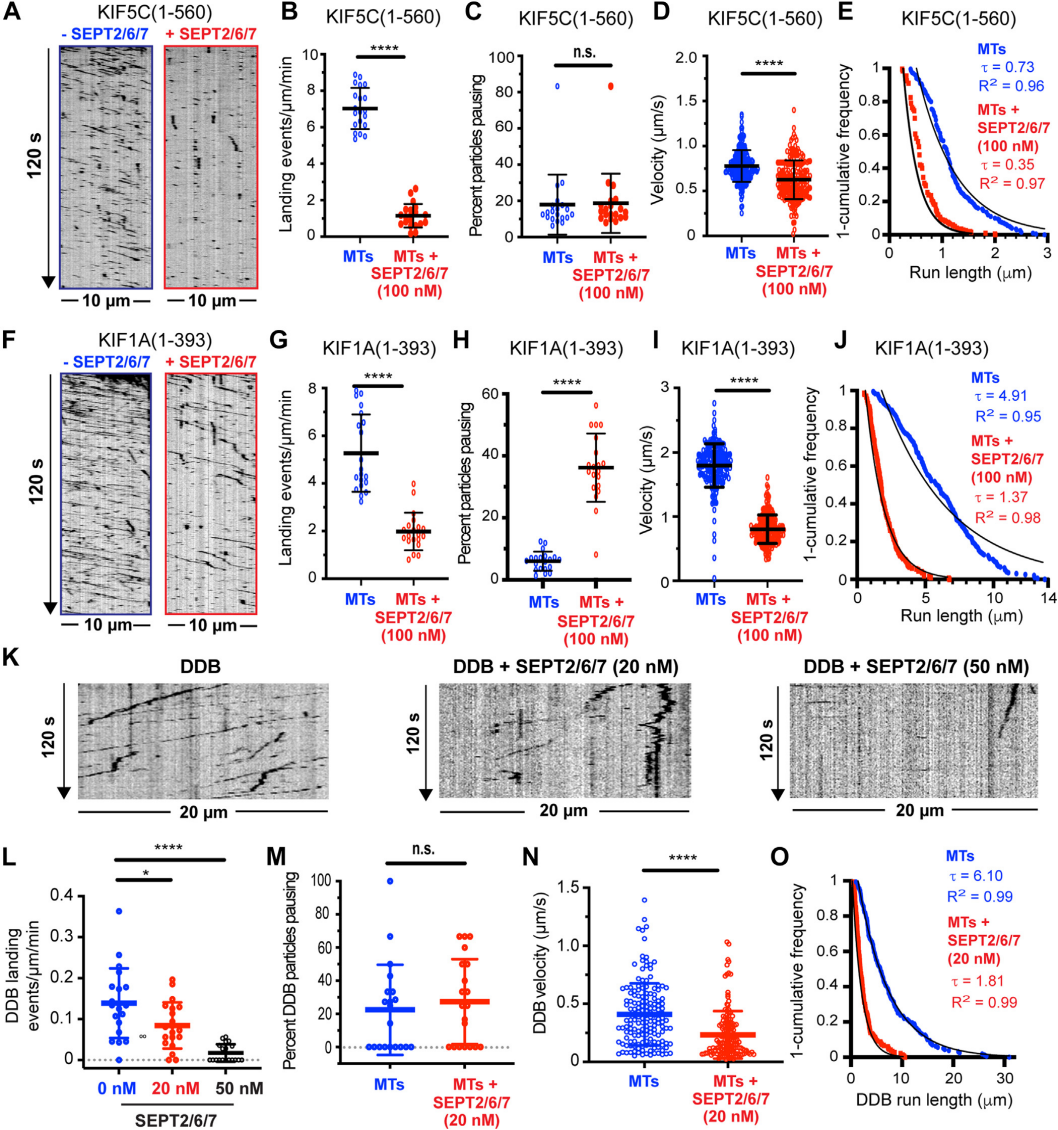
**Microtubule-associated SEPT2/6/7 complexes inhibit the motility of KIF5C, KIF1A, and DDB.***A*, kymographs show motile (*diagonal lines*) and stationary (vertical lines) KIF5C(1-560)-mCit on an uncoated microtubule (*left*) and a microtubule (*right*) coated with mCherry-SEPT2/6/7 (100 nM). *B*, mean (±S.D.) landing rates of KIF5C(1-560)-mCit on uncoated microtubules (7.03 ± 1.13 events/μm/min; *n* = 20 microtubules) and microtubules coated with 100 nM mCherry-SEPT2/6/7 (1.15 ± 0.65 events/μm/min; *n* = 20 microtubules). ∗∗∗∗*p* < 0.0001. *C*, mean (±S.D.) percentage of KIF5C(1-560)-mCit particles pausing on uncoated microtubules (17.94% ± 16.56%; *n* = 20 microtubules) and microtubules coated with 100 nM mCherry-SEPT2/6/7 (18.67% ± 16.33%; *n* = 20 microtubules). n.s., not significant (*p* > 0.05). *D*, mean (±S.D.) velocity of KIF5C(1-560)-mCit particles (*n* = 200) on uncoated microtubules (0.78 ± 0.18 μm/s) and microtubules coated with 100 nM of mCherry-SEPT2/6/7 (0.63 ± 0.22 μm/s). ∗∗∗∗*p* < 0.0001. *E*, one-cumulative distribution plot of the run lengths of KIF5C(1-560)-mCit particles (*n* = 200) on uncoated microtubules and microtubules with mCherry-SEPT2/6/7 (100 nM). Data were fit to one-phase exponential decay with a decay constant τ (run length), which is shown with the *R*^2^ fit value. The mean (±S.D.) run length values were 1.18 ± 0.52 μm and 0.64 ± 030 μm in the absence and presence of mCherry-SEPT2/6/7, respectively (*p* < 0.0001). *F*, kymographs show motile (*diagonal lines*) and stationary (*vertical lines*) KIF1A(1-393)-GCN4-3XmCit particles on an uncoated microtubule (*left*) and a microtubule (*right*), which was coated with mCherry-SEPT2/6/7 (100 nM). *G*, mean (±S.D.) landing rates of KIF1A(1-393)-GCN4-3XmCit on uncoated microtubules (5.27 ± 1.62 events/μm/min; *n* = 20 microtubules) and microtubules coated with 100 nM mCherry-SEPT2/6/7 (1.98 ± 0.79 events/μm/min; *n* = 20 microtubules). ∗∗∗∗*p* < 0.0001. *H*, mean (±S.D.) percentage of KIF1A(1-393)-GCN4-3XmCit particles pausing on uncoated microtubules (5.99% ± 3.07%; *n* = 20 microtubules) and microtubules coated with 100 nM mCherry-SEPT2/6/7 (36.19% ± 11.04%; *n* = 20 microtubules). ∗∗∗∗*p* < 0.0001. *I*, mean (±S.D.) velocity of KIF1A(1-393)-GCN4-3XmCit particles (*n* = 200) on uncoated microtubules (1.80 ± 0.34 μm/s) and microtubules coated with mCherry-SEPT2/6/7 (0.80 ± 0.22 μm/s). ∗∗∗∗*p* < 0.0001. *J*, one-cumulative distribution plot of the run lengths of KIF1A(1-393)-GCN4-3XmCit particles (*n* = 200) on uncoated microtubules and microtubules with mCherry-SEPT2/6/7 (100 nM). Data were fit to one-phase exponential decay with a decay constant τ (run length), which is shown with the *R*^2^ fit value. The mean (±S.D.) run length values were 6.06 ± 2.85 μm and 2.02 ± 1.13 μm in the absence and presence of mCherry-SEPT2/6/7, respectively (*p* < 0.0001). *K*, kymographs of DDB-GFP motility on an uncoated microtubule and microtubules coated with 20 nM and 50 nM mCherry-SEPT2/6/7. *L*, mean (±S.D.) landing rates of DDB-GFP particles on uncoated microtubules (0.14 ± 0.09 events/μm/min; *n* = 20 microtubules) and microtubules (*n* = 20) which were coated with 20 nM (0.08 ± 0.06 events/μm/min) and 50 nM mCherry-SEPT2/6/7 (0.02 ± 0.02 events/μm/min). ∗*p* = 0.04; ∗∗∗∗*p* < 0.0001. *M*, mean (±S.D.) percentage of DDB-GFP particles that pause on uncoated microtubules (22% ± 27%; *n* = 20) and microtubules (*n* = 20) coated with 20 nM mCherry-SEPT2/6/7 (27% ± 26%). n.s., not significant (*p* > 0.05). *N*, mean (±S.D.) velocity of DDB-GFP particles on uncoated microtubules (0.41 ± 0.27 μm/s; *n* = 159) and microtubules coated with 20 nM mCherry-SEPT2/6/7 (0.23 ± 0.20 μm/s; *n* = 150). ∗∗∗∗*p* < 0.0001. *O*, one-cumulative distribution plot of the run lengths of DDB-GFP particles on uncoated microtubules (*n* = 159) and microtubules coated with 20 nM mCherry-SEPT2/6/7 (*n* = 150). Data were fit to one-phase exponential decay with a decay constant τ (run length) which is shown with the *R*^2^ fit value. The mean (±S.D.) run lengths were 7.38 ± 5.65 μm and 2.57 ± 2.07 μm in the absence and presence of mCherry-SEPT2/6/7, respectively (*p* < 0.0001). Statistical analysis of data with normal and non-normal distributions was performed with student's t and Mann-Whitney U tests, respectively. A nonparametric one-way Welch ANOVA test was performed for multiple comparison groups, followed by a post hoc Dunnett T3 test for pairwise comparisons. DDB, dynein-dynactin-bicaudal D.

We first tested whether microtubule-associated SEPT2/6/7 affected the motility of the constitutively active motor domain of kinesin-1 KIF5C(1-560)-mCit (Fig. 1, *A*–*E*). SEPT2/6/7 drastically reduced the motile events of KIF5C(1-560)-mCit, the frequency of which was ∼15% of its value on uncoated microtubules (Fig. 1*B*). SEPT2/6/7 also diminished the velocity and run lengths of KIF5C(1-560)-mCit by ∼20% and ∼45%, respectively, but did not affect the rate of motor pausing (Fig. 1, *C*–*E*).

Next, we analyzed the motility of the dimeric kinesin-3 motor KIF1A(1-393)-GCN4-3xmCit (Fig. 1, *F*–*J*). Microtubule-associated SEPT2/6/7 complexes decreased the landing rates of KIF1A(1-393)-GCN4-3xmCit by ∼65%. This inhibitory effect was not as severe as observed for KIF5C(1-560)-mCit (Fig. 1*B*). However, SEPT2/6/7 had a stronger effect on the kinetics of KIF1A(1-393)-GCN4-3xmCit than KIF5C(1-560)-mCit. Velocity and run lengths decreased by ∼50% and ∼70%, respectively, and pausing increased by six-fold (Fig. 1, *H*–*J*). Thus, SEPT2/6/7 is an inhibitor of both KIF5C and KIF1A, blocking primarily the initial docking of KIF5C on microtubules and obstructing chiefly the stepping cycle of KIF1A along microtubules.

To test whether SEPT2/6/7 impacts dynein motility, we performed *in vitro* motility assays with the constitutively active GFP-tagged DDB (DDB-GFP). After coating microtubules with 50 to 100 nM mCherry-SEPT2/6/7, DDB-GFP microtubule binding was completely abolished (Fig. 1, *K* and *L*). We assayed DDB-GFP motility on microtubules after decoration with 20 nM mCherry-SEPT2/6/7, which did not block DDB-GFP motility. On these mCherry-SEPT2/6/7-coated microtubules, DDB-GFP landing rates decreased by ∼40% (Fig. 1*L*). Similar to the inhibition of kinesin motility, the velocity and run lengths of DDB-GFP were also reduced (Fig. 1, *N* and *O*). However, there was no increase in the pausing of DDB-GFP motors (Fig. 1*M*). Collectively, these data demonstrate that microtubule-associated SEPT2/6/7 complexes inhibit DDB-GFP binding to microtubules more potently than KIF5C and KIF1A.

### In microtubule-associated SEPT2/6/7/9 complexes, SEPT9 dampens the inhibition of KIF1A by SEPT2/6/7

The largest protomeric unit of septins is a dimer of tetramers, which consists of subunits from the SEPT2, SEPT6, SEPT7, and SEPT9 groups (33, 47). The isoform 1 of SEPT9 (SEPT9_i1) binds microtubules with higher affinity than SEPT2/6/7 (48, 49), and unlike the SEPT2/6/7 complex, SEPT9_i1 enhances KIF1A microtubule binding and motility (19). To test how SEPT9 and SEPT2/6/7 function in combination, we expressed in bacteria and purified a recombinant mCherry-tagged SEPT2/6/7/9 (Fig. S1*B*) as recently done for GFP-SEPT2/6/7/9 (50). Microtubules were fully decorated after incubation with mCherry-SEPT2/6/7/9 complexes at concentrations as low as 10 nM (Fig. S1, *G* and *H*), which resembled the uniform decoration of microtubules by SEPT9_i1 at concentrations of 10 nM (19). Similar to mCherry-SEPT2/6/7, microtubule binding was concentration-dependent, and the septin subunits SEPT6, SEPT7, and SEPT9 were also bound to microtubules with mCherry-SEPT2 (Fig. S1, *G*–*I*).

**Figure 2:**
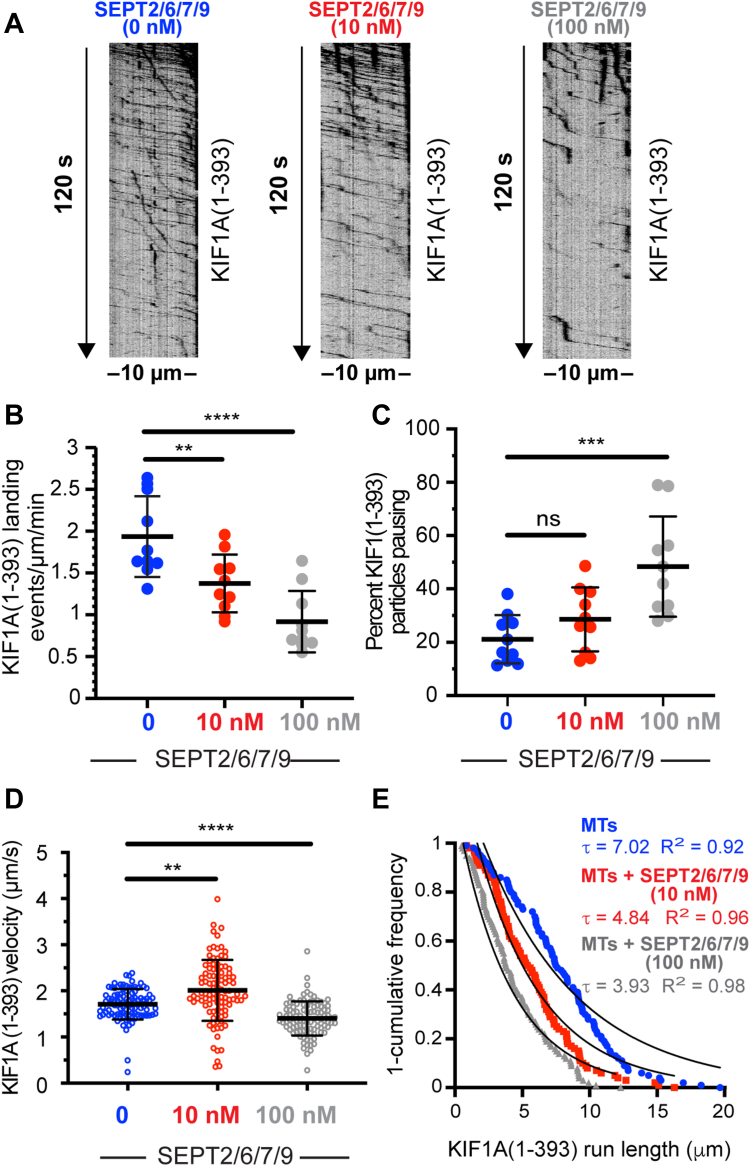
**In microtubule-associated SEPT2/6/7/9 complexes, SEPT9 dampens the inhibition of KIF1A(1-393) motility by SEPT2/6/7.***A*, kymographs show motile (*diagonal lines*) and stationary (*vertical lines*) KIF1A(1-393)-GCN4-3XmCit particles on an uncoated microtubule and microtubules which were coated with 10 nM or 100 nM mCherry-SEPT2/6/7/9. *B*, mean (±S.D.) landing rates of KIF1A(1-393)-GCN4-3XmCit on uncoated microtubules (1.94 ± 0.48 events/μm/min; *n* = 10 microtubules) and microtubules (*n* = 10) coated with 10 nM (1.37 ± 0.35 events/μm/min) or 100 nM mCherry-SEPT2/6/7/9 (0.92 ± 0.37 events/μm/min). ∗∗*p* = 0.008; ∗∗∗∗*p* < 0.0001. *C*, mean (±S.D.) percentage of KIF1A(1-393)-GCN4-3XmCit particles that pause on uncoated microtubules (21.09% ± 9.07%; *n* = 10 microtubules) and microtubules (*n* = 10) coated with 10 nM (28.56% ± 12%) or 100 nM mCherry-SEPT2/6/7/9 (48.35% ± 18.82%). n.s., not significant (*p* > 0.05); ∗∗∗∗*p* < 0.0001. *D*, mean (±S.D.) velocity of KIF1A(1-393)-GCN4-3XmCit particles (*n* = 100) on uncoated microtubules (1.71 ± 0.33 μm/s) and microtubules coated with 10 nM (2.01 ± 0.66 μm/s) or 100 nM mCherry-SEPT2/6/7/9 (1.40 ± 0.37 μm/s). ∗∗*p* = 0.002; ∗∗∗∗*p* < 0.0001. *E*, one-cumulative distribution plot of the run lengths of KIF1A(1-393)-GCN4-3XmCit particles (*n* = 100) on uncoated microtubules and microtubules coated with 10 nM or 100 nM mCherry-SEPT2/6/7/9. Data were fit to one-phase exponential decay with a decay constant τ (run length) which is shown with the *R*^2^ fit value. The mean (±S.D.) run length value on uncoated microtubules was 7.89 ± 3.88 μm, and the mean (±S.D.) run lengths on microtubules with 10 nM and 100 nM SEPT2/6/7/9 were respectively 5.84 ± 3.18 μm (*p* = 0.0003) and 4.44 ± 2.67 μm (*p* < 0.0001). Data were statistically analyzed with one-way ANOVA and a post hoc Dunnett test for multiple comparisons (*B* and *C*) or Kruskal–Wallis test with post hoc Dunn's test for multiple pairwise comparisons (*D* and *E*). SEPT9, septin 9.

To test how SEPT2/6/7/9 affects the motility of KIF1A(1-393)-GCN4-3xmCit, we decorated microtubules with 10 and 100 nM mCherry-SEPT2/6/7/9, the concentrations at which mCherry-SEPT9_i1 and mCherry-SEPT2/6/7 enhanced and inhibited KIF1A motility, respectively (Fig. 1, *F*–*J*; (19)). On microtubules decorated with 10 nM mCherry-SEPT2/6/7/9, the landing rates and run lengths of KIF1A decreased by ∼30% (Fig. 2, *A*, *B* and *E*). Strikingly, however, the velocity of KIF1A increased, and there was no effect on pausing (Fig. 2, *C* and *D*). On microtubules coated with 100 nM mCherry-SEPT2/6/7/9, the landing rate and run lengths of KIF1A decreased at levels that resembled in relative change the effects of SEPT2/6/7 (Figs. 1, *G* and *J* and 2, *B* and *E*). In contrast, the relative decrease in velocity was 3-fold more on SEPT2/6/7- than SEPT2/6/7/9-coated microtubules than uncoated microtubules (56% *versus* 18% reduction). KIF1A also paused more on SEPT2/6/7-coated microtubules—a 6-fold *versus* 2.3-fold increase in the percentage of paused motors (Figs. 1, *H* and *I* and 2, *C* and *D*). Taken together, these data indicate that SEPT9 dampens the inhibitory properties of the SEPT2/6/7 subunits and conversely, SEPT2/6/7 counteracts SEPT9 as a KIF1A enhancer, which results in a blended phenotype. Thus, the individual properties of septin subunits tune the regulatory function of a microtubule-associated septin complex.

We next tested how SEPT2/6/7/9 complexes impact the motility of KIF5C(1-560)-mCit and DDB-GFP. As predicted by the hindrance of KIF5C(1-560)-mCit motility by SEPT2/6/7 (Fig. 1, *A*–*E*) and SEPT9 (19), KIF5C(1-560)-mCit exhibited little and near complete lack of motility on microtubules decorated with 10 nM and 100 nM SEPT2/6/7/9, respectively (Fig. S2*A*). Similarly, the microtubule binding and motility of DDB-GFP decreased in a concentration-dependent manner after coating microtubules with 5 nM, 10 nM, and 20 nM mCherry-SEPT2/6/7/9 (Fig. S2, *B* and *C*). Because there was no motile DDB-GFP on microtubules which were decorated with 20 nM mCherry-SEPT2/6/7/9, we performed assays with microtubules coated with 5 nM and 10 nM mCherry-SEPT2/6/7/9. DDB-GFP velocity was slightly reduced, but run lengths diminished by over 50%, while pausing was not impacted (Fig. S2, *D*–*F*). This resembled the inhibitory profile of SEPT2/6/7, which also did not affect DDB-GFP pausing, possibly due to the ability of dynein to sidestep microtubule-bound obstacles by switching onto neighboring protofilaments (51).

### Sept5/7/11 permits KIF1A motility while inhibiting KIF5C and DDB

Given that the motility of KIF1A is differentially impacted by SEPT9 and SEPT2/6/7, we reasoned that septin complexes may have distinct regulatory properties based on the composition and identity of their subunits. To test this hypothesis, we generated a recombinant mCherry-tagged septin complex consisting of septins 5, 7, and 11 (Fig. S1*C*), which has been identified as a unique complex in neurons (52, 53). Incubation of taxol-stabilized microtubules with 50 nM mCherry-SEPT5/7/11 resulted in uniform and saturable coating of microtubules, which contained SEPT7 and SEPT11 along with mCherry-SEPT5 (Fig. S1, *J*–*L*).

**Figure 3:**
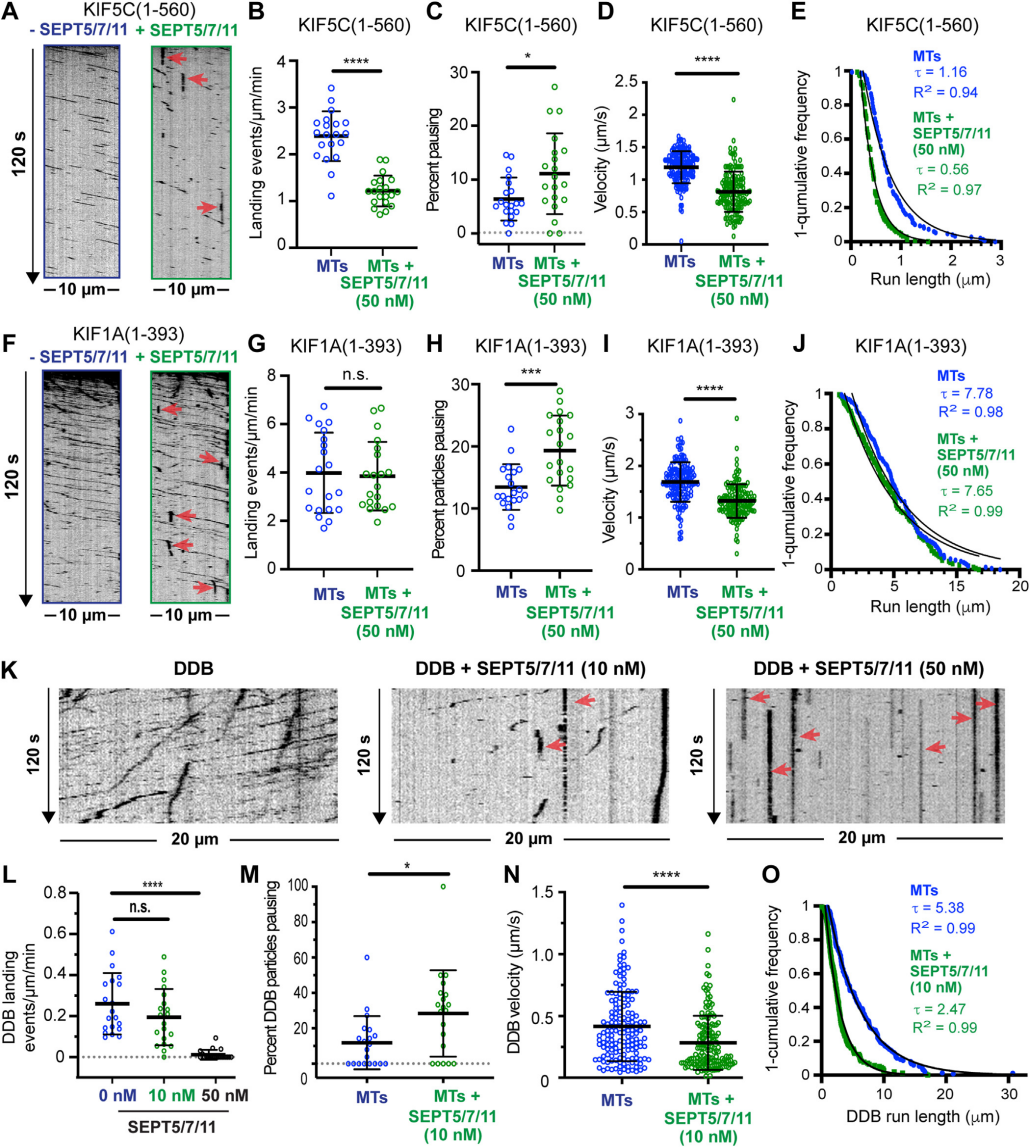
**Microtubule-associated SEPT5/7/11 complexes are permissive to KIF1A motility but inhibit KIF5C and DDB.***A*, kymographs show motile (*diagonal lines*) and stationary (*vertical lines*) KIF5C(1-560)-mCit on an uncoated microtubule (*left*) and a microtubule (*right*) which was coated with 50 nM mCherry-SEPT5/7/11. *Red arrows* point to KIF5C(1-560)-mCit motors, which remain immotile with no processive motility prior to dissociation (immotile particles). *B*, mean (±S.D.) landing rates of KIF5C(1-560)-mCit on uncoated microtubules (2.39 ± 0.53 events/μm/min; *n* = 20 microtubules) and microtubules coated with 50 nM mCherry-SEPT5/7/11 (1.22 ± 0.33 events/μm/min; *n* = 20 microtubules). ∗∗∗∗*p* < 0.0001. *C*, mean (±S.D.) percentage of KIF5C(1-560)-mCit particles pausing on uncoated microtubules (6.38% ± 4.00%; *n* = 20 microtubules) and microtubules coated with 50 nM mCherry-SEPT5/7/11 (11.10% ± 7.50%; *n* = 20 microtubules). ∗*p* = 0.02. *D*, mean (±S.D.) velocity of KIF5C(1-560)-mCit (*n* = 150) on uncoated microtubules (1.19 ± 0.25 μm/s) and microtubules coated with 50 nM of mCherry-SEPT5/7/11 (0.81 ± 0.31 μm/s). ∗∗∗∗*p* < 0.0001. *E*, one-cumulative distribution plot of the run lengths of KIF5C(1-560)-mCit particles (*n* = 150) on uncoated microtubules and microtubules with mCherry-SEPT5/7/11 (50 nM). Data were fit to one-phase exponential decay with a decay constant τ (run length), which is shown with the *R*^2^ fit value. The mean (±S.D.) run lengths were 1.58 ± 1 μm and 0.91 ± 0.48 μm in the absence and presence of mCherry-SEPT5/7/11, respectively (*p* < 0.0001). *F*, kymographs show motile (*diagonal lines*) and stationary (*vertical lines*) KIF1A(1-393)-GCN4-3XmCit particles on an uncoated microtubule (*left*) and a microtubule (*right*), which was coated with 50 nM mCherry-SEPT5/7/11. *Red arrows* point to KIF1A(1-393)-GCN4-3XmCit motors, which associate with a microtubule with no processive motility. *G*, mean (±S.D.) landing rates of KIF1A(1-393)-GCN4-3XmCit on uncoated microtubules (3.99 ± 1.66 events/μm/min; *n* = 20 microtubules) and microtubules (*n* = 20) coated with 50 nM mCherry-SEPT5/7/11 (3.85 ± 1.42 events/μm/min). n.s., not significant (*p* > 0.05). *H*, mean (±S.D.) percentage of KIF1A(1-393)-GCN4-3XmCit particles pausing on uncoated microtubules (13.44% ± 3.69%; *n* = 20 microtubules) and microtubules (*n* = 20) coated with 50 nM mCherry-SEPT5/7/11 (19.33% ± 5.63%; *n* = 20). ∗∗∗*p* = 0.0004. *I*, mean (±S.D.) velocity of KIF1A(1-393)-GCN4-3XmCit particles (*n* = 148) on uncoated microtubules (1.69 ± 0.38 μm/s) and microtubules coated with 50 nM mCherry-SEPT5/7/11 (1.32 ± 0.33 μm/s). ∗∗∗∗*p* < 0.0001. *J*, one-cumulative distribution plot of the run lengths of KIF1A(1-393)-GCN4-3XmCit particles (*n* = 148) on uncoated microtubules and microtubules with mCherry-SEPT5/7/11 (50 nM). Data were fit to one-phase exponential decay with a decay constant τ (run length), which is shown with the *R*^2^ fit value. The mean (±S.D.) run length values were 5.25 ± 2.59 μm and 4.68 ± 2.63 μm in the absence and presence of mCherry-SEPT2/6/7, respectively (*p* > 0.05). *K*, kymographs of DDB-GFP on an uncoated microtubule and microtubules which were coated with 10 nM and 50 nM mCherry-SEPT5/7/11. *Red arrows* point to DDB-GFP particles, which associate with microtubules with no processive motility (immotile particles). *L*, mean (±S.D.) landing rates of DDB-GFP particles on uncoated microtubules (0.26 ± 0.15 events/μm/min; *n* = 20 microtubules) and microtubules (*n* = 20) coated with 10 nM (0.19 ± 0.14 events/μm/min) and 50 nM mCherry-SEPT5/7/11 (0.01 ± 0.02 events/μm/min). n.s., not significant (*p* > 0.05); ∗∗∗∗*p* < 0.0001. *M*, mean (±S.D.) percentage of DDB-GFP particles that pause on uncoated microtubules (11.85% ± 14.98%; *n* = 20 microtubules) and microtubules (*n* = 20) which were coated with 10 nM mCherry-SEPT5/7/11 (28.37% ± 24.35%) ∗*p* = 0.01. *N*, mean (±S.D.) velocity of DDB-GFP particles on uncoated microtubules (0.42 ± 0.28 μm/s; *n* = 150) and microtubules coated with 50 nM mCherry-SEPT5/7/11 (0.28 ± 0.22 μm/s; *n* = 155). ∗∗∗∗*p* < 0.0001. *O*, one-cumulative distribution plot of the run lengths of DDB-GFP (*n* = 150–155) on uncoated microtubules and microtubules coated with mCherry-SEPT5/7/11 (50 nM). Data were fit to one-phase exponential decay with a decay constant τ (run length), which is shown with the *R*^2^ fit value. The mean (±S.D.) run length values were 6.29 ± 4.86 μm (*n* = 150) and 3.14 ± 2.80 μm in the absence and presence of mCherry-SEPT5/7/11, respectively (*p* < 0.0001). Statistical analysis of data with normal and non-normal distributions was performed with student's t and Mann-Whitney U tests, respectively. A nonparametric one-way ANOVA Kruskal–Wallis test was performed for multiple comparison groups, followed by a post hoc Dunn's test for pairwise comparisons. DDB, dynein-dynactin-bicaudal D.

We analyzed the motility of KIF5C(1-560)-mCit on microtubules coated with 50 nM mCherry-SEPT5/7/11 and found that landing rates diminished by ∼50% (Fig. 3, *A* and *B*). The velocity and run lengths of microtubule-bound KIF5C(1-560)-mCit decreased, and there was a 1.7-fold increase in the percentage of motors that paused (Fig. 3, *C*–*E*). Strikingly, however, the landing rates of KIF1A(1-393)-GCN4-3xmCit were not impacted (Fig. 3, *F* and *G*). Additionally, SEPT5/7/11 had modest effect on the pausing and velocity of KIF1A(1-393)-GCN4-3xmCit and no effect on run lengths (Fig. 3, *H* and *I*). Thus, in contrast to SEPT2/6/7, SEPT5/7/11 is largely permissive to the motility of KIF1A.

Analysis of DDB-GFP motility on microtubules coated with mCherry-SEPT5/7/11 revealed a strong inhibition. On microtubules coated with mCherry-SEPT5/7/11 at concentrations over 50 nM, there was no DDB-GFP motility (Fig. 3, *K* and *L*). After decoration of microtubules with 10 nM mCherry-SEPT5/7/11, which permitted DDB-GFP landing, we found that DDB-GFP velocity and run lengths diminished, and there was a 2.5-fold increase in pausing (Fig. 3, *M*–*O*). Although the inhibition of DDB-GFP motility by SEPT5/7/11 resembled SEPT2/6/7, the enhanced rates of pausing indicate that the underlying mechanism is different and DDB-GFP cannot bypass SEPT5/7/11. This was also consistent with an increase of immotile microtubule-bound DDB-GFP particles, which did not undergo processive motility (Fig. 3*K*, red arrows). These data show that microtubule-associated SEPT5/7/11 differs from SEPT2/6/7 in permitting KIF1A motility and DDB binding to microtubules. Strikingly, KIF1A emerges as a kinesin motor which is differentially modulated by different septin subunits and combinations thereof, which can enhance (SEPT9), inhibit (SEPT2/6/7), or permit KIF1A motility without major interference (SEPT5/7/11).

### Sept5/7/11 promotes DDB and kinesin tethering to microtubules in a septin complex-specific manner

**Figure 4:**
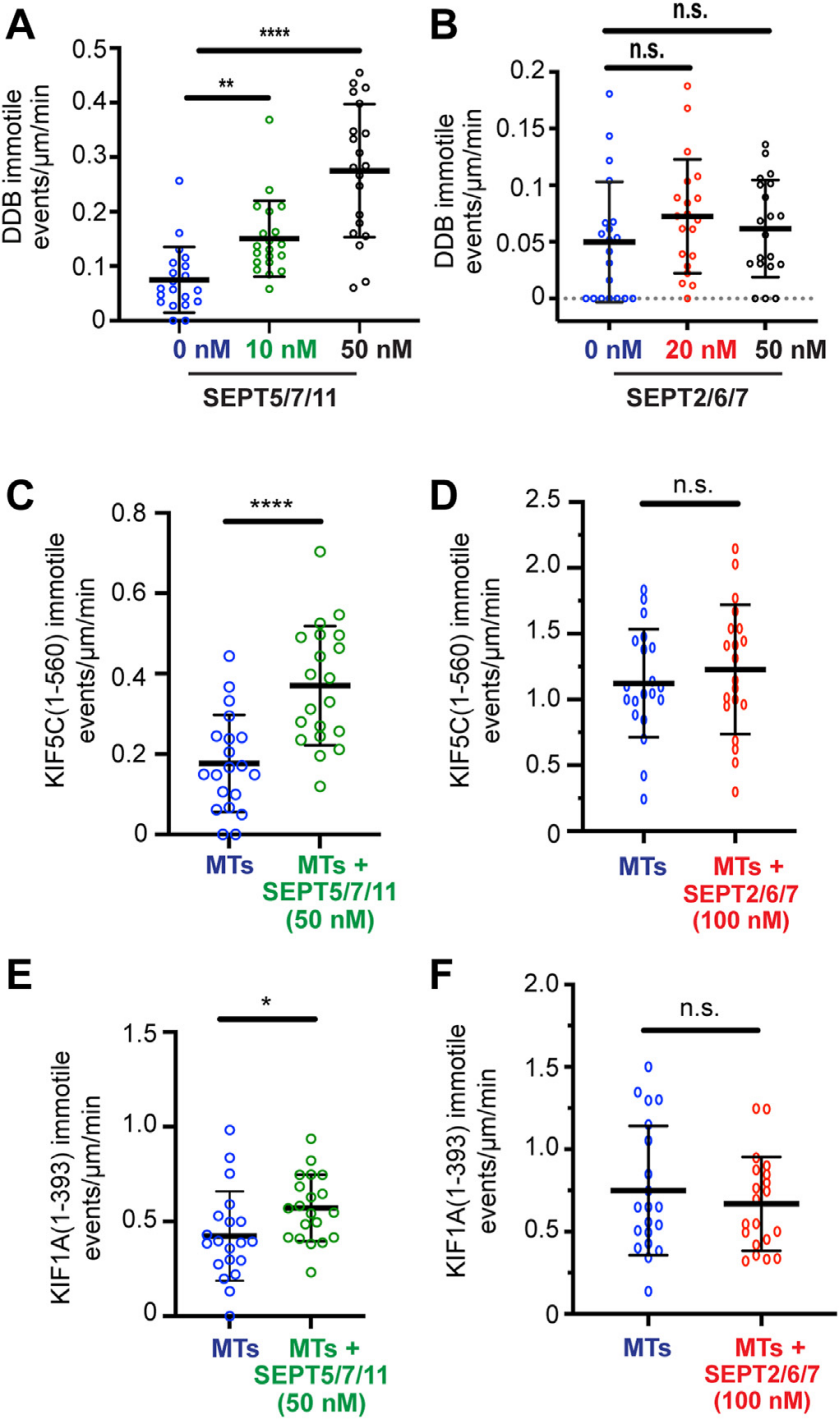
**SEPT5/7/11 promotes DDB and kinesin tethering to microtubules.***A*, mean (±S.D.) number of immotile DDB-GFP events per micrometer of uncoated microtubules (0.07 ± 0.06 events/μm/min; *n* = 20 microtubules) and microtubules (*n* = 20) coated with 10 nM (0.15 ± 0.07 events/μm/min) or 50 nM mCherry-SEPT5/7/11 (0.28 ± 0.12 events/μm/min). ∗∗*p* = 0.006; ∗∗∗∗*p* < 0.0001. *B*, mean (±S.D.) number of immotile DDB-GFP events per micrometer of uncoated microtubules (0.05 ± 0.05 events/μm/min; *n* = 20 microtubules) and microtubules (n = 20) coated with 20 nM (0.07 ± 0.05 events/μm/min) or 50 nM mCherry-SEPT2/6/7 (0.06 ± 0.04 events/μm/min). n.s., not significant (*p* > 0.05). *C*, mean (±S.D.) number of immotile KIF5C(1-560)-mCit particles per micrometer of uncoated microtubules (0.18 ± 0.12 events/μm/min; *n* = 20 microtubules) and microtubules (*n* = 20) coated with 50 nM mCherry-SEPT5/7/11 (0.37 ± 0.15 events/μm/min). ∗∗∗∗*p* < 0.0001. *D*, mean (±S.D.) number of immotile KIF5C(1-560)-mCit events per micrometer of uncoated microtubules (1.12 ± 0.41 events/μm/min; *n* = 20 microtubules) and microtubules (*n* = 20) coated with 100 nM mCherry-SEPT2/6/7 (1.23 ± 0.49 events/μm/min). n.s., not significant (*p* > 0.05). *E*, mean (±SEM) number of immotile KIF1A(1-393)-GCN4-3XmCit events per micrometer of uncoated microtubule (0.42 ± 0.24 events/μm/min; *n* = 20 microtubules) and microtubules (*n* = 20) coated with 50 nM mCherry-SEPT5/7/11 (0.57 ± 0.18 events/μm). ∗*p* = 0.03. *F*, mean (±SEM) number of immotile KIF1A(1-393)-GCN4-3XmCit events per micrometer of uncoated microtubule (0.75 ± 0.39 events/μm/min; *n* = 20 microtubules) and microtubules (*n* = 20) coated with 50 nM mCherry-SEPT2/6/7 (0.67 ± 0.29 events/μm/min). n.s., not significant (*p* > 0.05). Statistical analysis of data with normal and non-normal distributions was performed with student's t and Mann-Whitney U tests, respectively. A nonparametric one-way ANOVA Kruskal–Wallis test was performed for multiple comparison groups, followed by a post hoc Dunn's test for pairwise comparisons. DDB, dynein-dynactin-bicaudal D.

In assays of DDB-GFP motility, we observed numerous DDB-GFP particles landing and remaining static on microtubules with mCherry-SEPT5/7/11 (Fig. 3*K*, red arrows). We quantified the frequency of these immotile events as number of particles that associate with microtubules for >600 ms without undergoing any processive motion. We found that the frequency of DDB-GFP immotile events increased with higher densities of microtubule-bound SEPT5/7/11 (Fig. 4*A*)—that is, microtubules coated with increasing concentrations (0–50 nM) of mCherry-SEPT5/7/11 (Fig. S1*J*)—and inversely correlated with the landing rates of DDB-GFP, which decreased in a dose-dependent manner (Fig. 3*L*). This DDB behavior was unique to SEPT5/7/11-coated microtubules. On microtubules coated with increasing concentrations of mCherry-SEPT2/6/7 or mCherry-SEPT2/6/7/9, there was no increase in the frequency of immotile DDB-GFP particles in spite of a concentration-dependent reduction in the landing rate of DDB-GFP (Figs. 4*B* and S3*A*).

We next inquired whether SEPT5/7/11 impacts the fraction of immotile kinesins. We analyzed the frequency of immotile events of KIF5C(1-560)-mCit and KIF1A(1-393)-GCN4-3xmCit (Fig. 3, *A* and *F*, red arrows). We found a two-fold increase in the immotile events of KIF5C(1-560)-mCit on microtubules coated with mCherry-SEPT5/7/11, while there was no effect on microtubules coated with mCherry-SEPT2/6/7 (Fig. 4, *C* and *D*). Similarly, SEPT5/7/11 enhanced the immotile events of KIF1A(1-393)-GCN4-3xmCit, but SEPT2/6/7 and SEPT2/6/7/9 had no effect (Figs. 4, *E* and *F* and S3*B*). Collectively, these data show that SEPT5/7/11 complexes can tether in place DDB and kinesin motors upon attaching to microtubules, which might be functionally critical for the microtubule anchoring of membrane organelles.

### Neuronal Golgi ribbons align with SEPT5-coated microtubules and depend on SEPT5 for their somatodendritic localization

**Figure 5:**
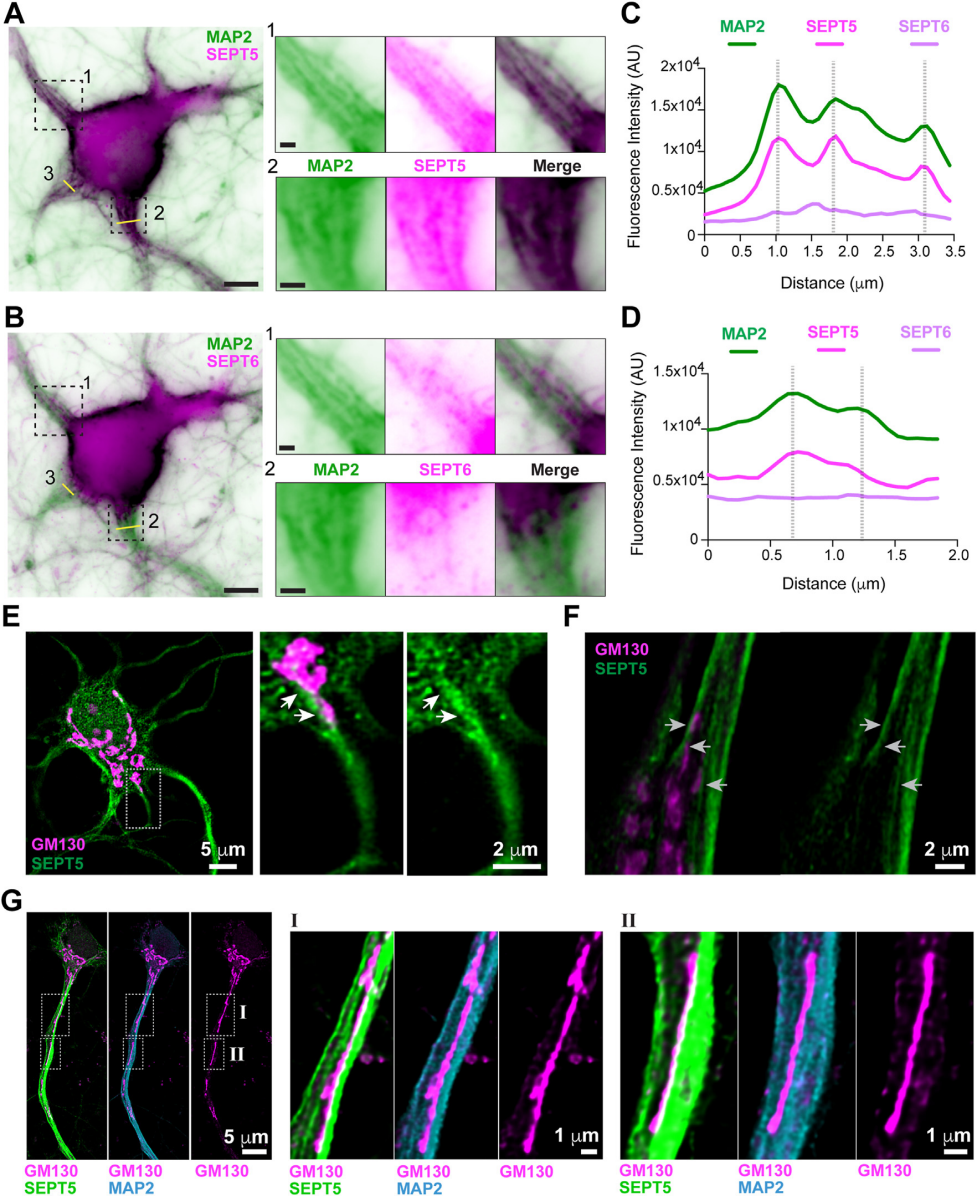
**Golgi membranes align along SEPT5-coated microtubules in neuronal dendrites.***A* and *B*, representative TIRF microscopy images of a primary rat hippocampal neuron (DIV14), which was stained with antibodies to SEPT5 (*A*; inverted forest *green*), SEPT6 (*B*; inverted forest *green*), and MAP2 (inverted *magenta*). The same cell is stained and shown in *panels A* and *B*, and the MAP2 image is reused. Areas in *dashed rectangles* (1, 2) are shown in higher magnification. *Yellow lines* 2 and 3 designate the linescan regions in *C* and *D*, respectively. Scale bars represent 5 μm and 1 μm (magnified regions). *C* and *D*, plot profiles of the fluorescence intensities of SEPT5, SEPT6, and MAP2 across the lines 2 (*C*) and 3 (*D*) shown in *panels A* and *B*, respectively. *Dashed vertical lines* mark peaks of fluorescence that correspond to microtubule bundles. *E* and *F*, deconvolution wide-field microscopy images of embryonic rat hippocampal neurons (DIV10) stained for GM130 (*magenta*) and SEPT5 (*green*). Arrows point to GM130-labeled Golgi membranes, which are docked on SEPT5 filaments at the base of MAP2-positive (not shown) dendrites. Scale bars represent 5 μm and 2 μm (magnified regions). *G*, super-resolution confocal microscopy of a primary embryonic rat hippocampal neuron (DIV10), which was stained for SEPT5 (*green*), MAP2 (*cyan*), and GM130 (*magenta*) shows tubular Golgi membranes deployed into the principal dendrite. Regions outlined with *dashed lines* (I, II) are shown in higher magnification. Scale bars represent 5 μm and 1 μm (magnified regions). MAP, microtubule-associated protein; TIRF, total internal reflection fluorescence.

The differential effects of SEPT2/6/7 and SEPT5/7/11 on kinesin and DDB motility suggest that these septin complexes may have distinct physiological functions. We explored this possibility by probing for the localization of SEPT2/6/7 and SEPT5/7/11 in the microtubule network of hippocampal neurons, where SEPT5/7/11 was originally identified as a biochemically distinct complex (52). Primary embryonic rat hippocampal neurons, which had developed axonal and dendritic processes after 14 days in culture (DIV14), were stained with antibodies against SEPT5 and SEPT6 as proxy subunits for SEPT5/7/11 and SEPT2/6/7, respectively. To better resolve microtubules and septins in the somatodendritic compartment, we extracted neurons prior to fixation and costained for the MAP2 which decorates the microtubule bundles of dendrites. Using shallow angle TIRF microscopy, we observed SEPT5 colocalizing with MAP2-labeled microtubules in the somato-proximal segments of dendritic shafts (Figs. 5, *A* and *B* and S4*A*). SEPT6, however, was largely absent from the long MAP2-labeled tracks and consisted of short filaments and puncta (Figs. 5, *C* and *D* and S4*B*). Thus, SEPT5-containing complexes are enriched on dendritic microtubule bundles, which lack SEPT6.

We next probed for the localization of the Golgi complex with respect to the dendritic SEPT5-coated microtubules. We focused on the neuronal Golgi, because following axon initiation, it polarizes toward the base of the presumptive apical dendrite and Golgi ribbons align along the microtubules of the somato-proximal segment of dendrites—a phenomenon known as Golgi deployment that precedes the formation and dispatch of Golgi outposts (54, 55, 56, 57). How Golgi ribbons assume their polarized localization is poorly understood. We reasoned that SEPT5/7/11 complexes might play a role because of the enrichment of SEPT5 on somato-proximal microtubules and the *in vitro* tethering of DDB motors to SEPT5/7/11-coated microtubules. Of note, DDB associates with Golgi membranes and is critical for Golgi morphology and localization (58, 59, 60).

Using wide-field microscopy with advanced optical clearing and super-resolution confocal microscopy, we imaged the localization of the Golgi marker protein GM130 with respect to SEPT5 in rat hippocampal neurons. We observed Golgi stacks anastomosing with the somato-proximal ends of SEPT5 tracks, which extend into the dendritic shaft (Fig. 5, *E* and *F*). In confocal 3D image stacks, 66 ± 3% (n = 12 neurons) of Golgi volume overlapped with SEPT5, and the Manders overlap coefficient was 0.55 ± 0.11. In dendrites with Golgi ribbons, which are deployed well into the shaft, there was a tight coalignment of GM130-labeled tubules with SEPT5-enriched tracks, which appeared to be zippered together (Fig. 5*G*). Golgi ribbons made no contacts with SEPT6 filaments in dendritic shafts, but occasional end-to-end and orthogonal contacts were observed in the cell body (Fig. S4, *B* and *C*).

**Figure 6:**
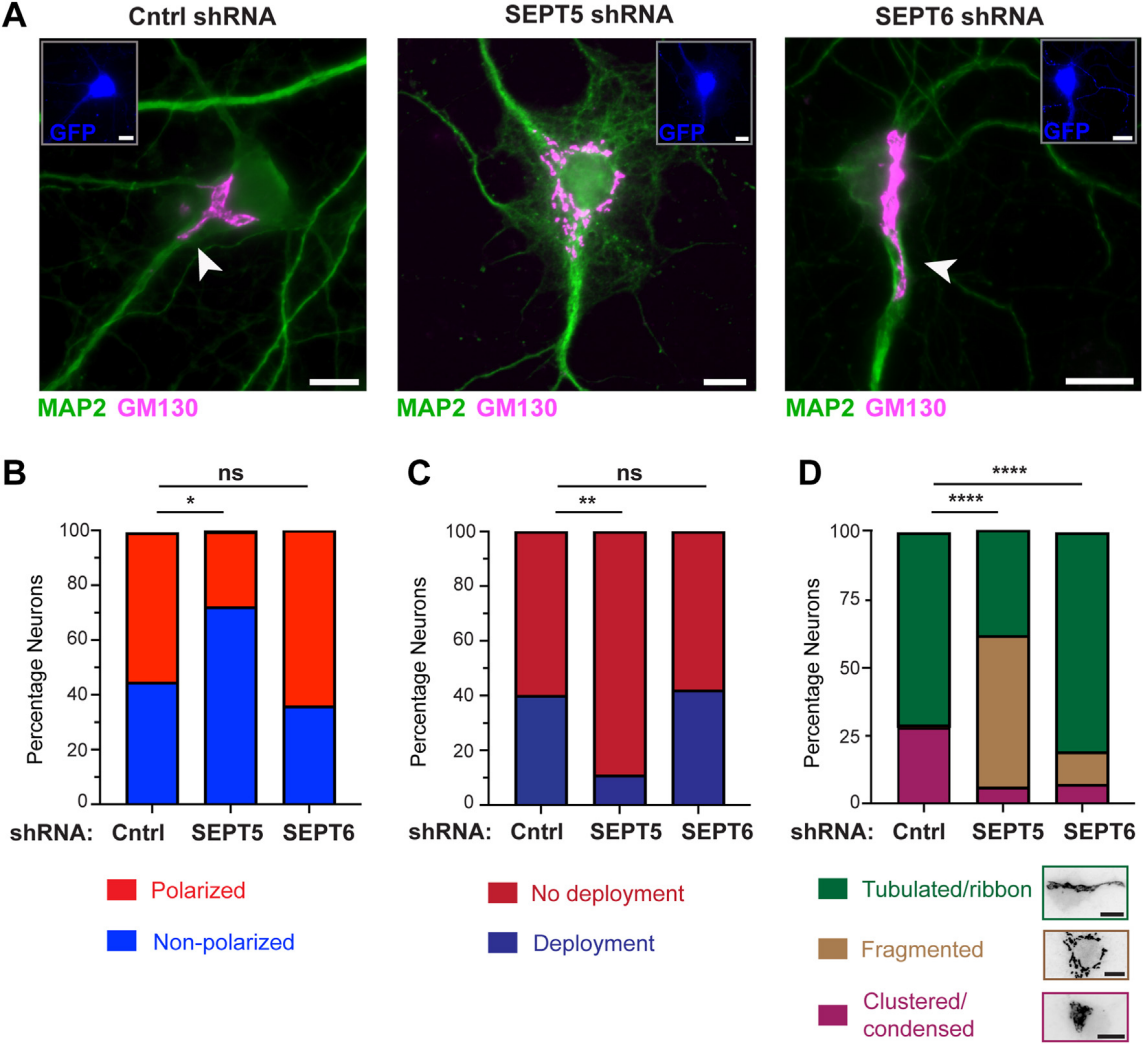
**Golgi localization and morphology are disrupted by SEPT5 depletion.***A*, images show Golgi (GM130; *magenta*) localization with respect to MAP2 (*green*)-labeled dendrites in primary embryonic rat hippocampal neurons (DIV14) after a 4-day transfection with plasmids that express GFP (*blue*) and shRNAs. *Arrowheads* point to Golgi tubules which are deployed into dendrites. Scale bars represent 10 μm. *B*–*D*, bar graphs show quantification of Golgi polarization (*B*), which was scored as Golgi localization at the base of a single dendrite, and Golgi deployment into a dendrite (*C*; Golgi tubules stretching along the beginning of the dendritic shaft). Golgi morphology (*D*) was categorized into clustered/condensed, fragmented, or tubulated with ribbon-like appearance. Quantifications were performed in rat hippocampal neurons (DIV14) after 3 days of transfection with control shRNA (*n* = 75) and shRNAs against SEPT5 (*n* = 36) and SEPT6 (*n* = 99). Statistical analysis for pairwise comparisons was done with the chi square test (n.s., not significant; ∗*p* < 0.05; ∗∗*p* < 0.01; ∗∗∗∗*p* < 0.0001). The chi square group test result for Golgi polarity was *Χ*^2^ (2, *N* = 30) = 13.67, *p* = 0.001076. The chi square group test result for Golgi deployment was *Χ*^2^ (2, *N* = 30) = 11.94, *p* = 0.00251 and for Golgi morphology, *Χ*^2^ (4, *N* = 45) = 69.08, *p* = 0.00001. Scale bars represent 10 μm. MAP, microtubule-associated protein.

We next tested whether SEPT5 contributes to the localization and morphology of the neuronal Golgi complex. We analyzed Golgi localization in rat DIV14 hippocampal neurons that co-expressed GFP and SEPT5- or SEPT6-specific shRNAs, which previously impacted ankyrin G localization in the axon initial segment of rat DIV13-17 hippocampal neurons (61). We limited our analysis to neurons with 4′,6-diamidino-2-phenylindole (DAPI)-stained nuclei that lacked condensed chromatin, a sign of apoptosis which disrupts Golgi integrity (62). SEPT5 knockdown, which also decreases SEPT7 levels (63, 64), reduced the percentage of neurons with the Golgi oriented toward a single dendrite by ∼50%, while SEPT6 did not have an effect (Fig. 6, *A* and *B*). In addition, SEPT5 depletion reduced dramatically the percentage of neurons with deployed Golgi ribbons, while SEPT6 had no effect (Fig. 6*C*). We also assessed the overall morphology of the Golgi complex in the cell bodies of neurons, which had a condensed, tubulated ribbon-like, or fragmented morphology (Fig. 6*D*). SEPT5 depletion markedly reduced the percentage of neurons with condensed and tubulated morphology, and over 50% of neurons contained a fragmented Golgi (Fig. 6*D*). In SEPT6-depleted neurons, there was a small increase of the percentage of neurons with tubulated and fragmented Golgi (Fig. 6*D*), which may stem from the contacts of SEPT6 with Golgi membranes in the soma (Fig. S4*C*). Given that SEPT5/7/11 immobilizes dynein *in vitro*, we examined dynein localization with respect to GM130-labeled Golgi ribbons and stacks. Upon staining with an antibody against dynein intermediate chain, dynein distribution resembled the pattern of Golgi localization and included dynein presence on deployed Golgi ribbons (Fig. S5*A*, arrowheads). SEPT5 knockdown reduced the levels of dynein intermediate chain at GM130-labeled Golgi stacks, which could be due to loss of dynein tethering on underlying microtubules (Fig. S5, *B*–*D*). Collectively, these data show that microtubule-associated SEPT5-containing complexes have a distinct function in neuronal Golgi localization and organization, which is in agreement with the *in vitro* immobilization of dynein and kinesin on SEPT5/7/11-coated microtubules.

## Discussion

In the microtubule network, spatial control of membrane traffic and organelle positioning is largely mediated by MAPs and the post-translational modifications (PTMs) of microtubules (4, 5, 6, 7). Subsets of microtubules have unique combinations of MAPs and PTMs, which determine the type of motors that bind a microtubule and their motile behaviors (3, 8, 10, 65). Recent work has begun to shed light on the selective regulation of kinesin and dynein motility by distinct MAPs, some of which can function as either suppressors or enhancers of motor motility (12, 13, 14, 15, 19, 20, 21). In combination with microtubule PTMs, these MAPs determine the intracellular routes and positions of various membrane cargos.

We previously identified SEPT9 as a MAP that differentially regulates motility of kinesins KIF5C, KIF1A, and KIF17 (19). This finding raised the possibility that other members of the septin family selectively regulate kinesin and dynein motility (42). Here, we sought to explore this hypothesis by testing how two different septins complexes (SEPT2/6/7, SEPT5/7/11) impact the kinesins KIF5C and KIF1A and the dynein motor complex DDB. Moreover, we tested whether septin subunits influence the collective property of their respective complexes by examining how the SEPT2/6/7/9 complex impacts KIF1A motility, which is enhanced by SEPT9 but inhibited by SEPT2/6/7 (19). This is an important question of broader significance for septin biology as it remains poorly understood how the function of septin complexes is determined by their individual subunits (66). In rat hippocampal neurons, the existence of a SEPT5/7/11 complex has been independently confirmed (52, 53, 63), but it is unclear whether it has distinct or overlapping functions with SEPT2/6/7. SEPT2 and SEPT6 can replace SEPT5 and SEPT11, respectively, in the SEPT5/7/11 complex, though SEPT2 expression is reportedly lower than the other septin subunits (63, 67, 68). In the neuronal microtubule network, the localization and functions of these septin complexes have not been explored. Because microtubule lattices are coated with combinations of MAPs, heteromeric septin complexes can serve as a model for how combinations of MAPs regulate kinesin and dynein motility along the microtubule lattice.

Our results reveal that the microtubule-associated SEPT5/7/11 complex has two fundamental differences from SEPT2/6/7. In contrast to SEPT2/6/7, which strongly inhibits the microtubule binding and motility of KIF5C, KIF1A, and DDB, SEPT5/7/11 is first permissive to the microtubule binding and motility of KIF1A and second, enhances the fraction of microtubule-tethered motors that remain immotile without processive movement. The latter anchoring-like effect was most pronounced for DDB, which exhibited no motility while bound on microtubules coated with a SEPT5/7/11 density that allowed kinesin movement, albeit at lower levels. In contrast to SEPT5/7/11, microtubule-associated SEPT2/6/7 and SEPT2/6/7/9 inhibited microtubule-DDB binding and thus, there was no DDB dwelling on microtubules.

Tethering of motors to the microtubule ends and lattice is critical for cellular structures such as motile cilia and flagella and the positioning of the spindle during mitosis (69, 70, 71, 72). DDB motility is inhibited on microtubules with detyrosinated α-tubulin, and dynein accumulates at microtubule minus ends (73, 74). Previous studies suggested that MAPs such as MAP2, MAP4, tau, and MAP7 inhibit dynein motility (13, 14, 21, 22, 23, 24, 25), but some of the evidence is controversial and it is unclear whether these MAPs can anchor cytoplasmic dynein to the microtubule lattice. Mechanistically, SEPT5/7/11 may tether DDB in a similar manner to how MAP7 keeps KIF5 tethered to the microtubule lattice while occluding the motor domain of KIF5 from stepping due to mutually exclusive microtubule-binding sites (14). In support of this possibility, we found a SEPT5/7/11 concentration-dependent increase in DDB immobilization, which occurs concomitantly with a concentration-dependent decrease in processive movement.

Dynein immobilization and inhibition of its motility on microtubules is hard to achieve owing to the ability of dynein to maneuver around MAPs by side-stepping and/or moving backwards (22, 51, 75). Septins appear to be potent inhibitors of DDB, but they differ mechanistically with SEPT2/6/7 and SEPT2/6/7/9 blocking microtubule binding and SEPT5/7/11 immobilizing dynein upon microtubule attachment. Independently of the mechanism, this inhibition is stronger for DDB than KIF5C and KIF1A, which are motile on microtubules coated with septin concentrations that do not permit DDB motility. On intracellular microtubules, we posit that these septin complexes may bias membrane traffic toward microtubule plus ends, being more restrictive to dynein- than kinesin-driven motility. Moreover, the motility of KIF1A-bound cargo might be selectively favored on microtubules with SEPT5/7/11 and SEPT2/6/7/9, which are more permissive to KIF1A than DDB and KIF5C. Thus, septin complexes may impact bidirectional transport, resolving the tug-of-war between groups of cargo-bound motors with opposing directionalities.

The SEPT2/6/7/9 complex is less potent than SEPT2/6/7 in reducing the velocity of KIF1A and enhancing its pausing, which is due to the presence of SEPT9, a KIF1A enhancer. Addition of SEPT9 to SEPT2/6/7 dampens the inhibitory effects of SEPT2/6/7 on motile KIF1A motors, but it does not appear to reduce the inhibition of KIF1A landing on microtubules by SEPT2/6/7. At the microtubule-bound density of SEPT9, which previously enhanced KIF1A motility, SEPT2/6/7/9 caused a modest decrease in the landing rates and run lengths of KIF1A and slightly enhanced KIF1A velocity. Thus, SEPT2/6/7 counteracts SEPT9 as an enhancer of KIF1A motility. We posit that this combinatorial blending of inhibitory and enhancing effects may underlie the modulation of kinesin and dynein motility on microtubule lattices, which are coated by combinations of different MAPs.

The distinct properties of SEPT5/7/11 and in particular the microtubule tethering of DDB led us to examine whether SEPT5/7/11 has a septin-specific role in the positioning of the Golgi complex in neurons. We focused on the Golgi complex because its morphology and distribution are largely dependent on dynein (76). Additionally, septins have been previously implicated in Golgi organization and post Golgi membrane traffic in non-neuronal cells (77, 78). We found Golgi stacks and ribbons localizing along SEPT5-coated microtubules at the somato-proximal segments of neuronal dendrites. SEPT6 was absent from these microtubule tracks, which indicated that SEPT5/7/11 may have a septin-specific function in Golgi association with the microtubules of proximal dendrites. Indeed, SEPT5 depletion resulted in the loss of Golgi polarization and Golgi ribbon localization along the basal segments of dendrites, while SEPT6 depletion did not have an effect. These data indicate that SEPT5/7/11 complexes promote tethering of Golgi membranes on a subset of microtubules that span the somato-proximal segment of dendrites.

*In vitro* tethering of DDB to SEPT5/7/11-coated microtubules suggests that SEPT5/7/11 immobilizes Golgi-associated dynein motors on somatodendritic microtubules. Golgi membranes associate with dynein *via* BICD adapters, and thus neuronal Golgi localization may involve regulation of DDB motility by SEPT5/7/11 (58, 59, 60). In parallel to tethering of Golgi-bound DDB to microtubules, SEPT5/7/11 may prevent association of Golgi-bound kinesin-1 motors with microtubules. Inhibition of kinesin-1 is critical for the dendritic localization of Golgi outposts (79), and inhibition of the motor domain of KIF1C prevents Golgi fragmentation (80). We posit that microtubule-associated SEPT5/7/11 tethers and/or constrains the motility of Golgi stacks and ribbons, which is conducive to the homotypic fusions that maintain the Golgi ribbon morphology (81, 82). In the absence of a restricted motility, Golgi membranes would haphazardly scatter throughout the soma, leading to a dispersed morphology of Golgi in SEPT5-depleted cells.

In sum, our results have revealed that microtubule-associated complexes can differentially and selectively modulate the motility of kinesin and dynein-dynactin motors. Importantly, this differential regulation stems from the properties of the individual septin subunits, which function combinatorially in modulating the microtubule attachment, dwelling stepping of microtubule motors. The modularity of septin assembly and the existence of multiple septin subunits with various isoforms point to a septin code, which functions in synergy with the tubulin and MAP codes for the spatial control of membrane traffic and organelle positioning.

## Experimental procedures

### Plasmids

For bacterial expression and purification of mCherry-SEPT2/6/7, the plasmids pET28a-mCherry-SEPT2 and pnCS SEPT6/7-Strep(+1-57 bp SEPT7 N-term) were used and described previously (44). For the expression of SEPT2/6/7/9, we used the plasmid pnCS_SEPT7_SEPT9_i1-TEV-Strep (addgene plasmid 174498), which was described previously (50), and the plasmid pnEA-vH_His-TEV-SEPT2-mCherry_SEPT6. The latter was constructed by replacing the msfGFP sequence of pnEA-vH_His-TEV-SEPT2-msfGFP_SEPT6 (Addgene plasmid # 174498) with mCherry (Iv *et al.*, 2021). This was done by amplifying the mCherry sequence from the plasmid mCherry-N1 (Clontech) and inserting into the BmbNI/XbaI sites of pnEA-vH_His-TEV-SEPT2-msfGFP_SEPT6 using the overlapping primers 5′GACGGCGACGGCGGGGCTCTCGGCCACGCAATGGTGAGCAAGGGCGAGGA3′ and 5′ GATCTCCTAGGGCTAGCTCTAGATTAGGACTACTTGTACAGCTCGTCCATG 3′ by Gibson reaction. For the expression and purification of SEPT5/7/11, we constructed a pET28a-mCherry-SEPT5 plasmid by PCR amplifying mCherry-SEPT5 from the plasmid mCherry-C1 SEPT5 using the primers 5′-AAAAAAGAATTCATGGTGAGCAAG-3′ and 5′-TTTTTTCTCGAGTCACTGGTCCTG-3′ and inserting into the EcoRI/XhoI sites of the pET28a(+) vector. The mCherry-C1-SEPT5 plasmid was made by PCR amplifying SEPT5 (human SEPT5 isoform 1) from the plasmid GST-SEPT5 WT (addgene # 27269) and inserting it into the mCherry-C1 vector (Clontech). In addition, we constructed the plasmid pET-Duet-1_SEPT11-7-Strep using the cloning services of Biomatik. The sequences encoding for human SEPT11 (isoform 1) and SEPT7-Strep tag (human SEPT7 isoform 1) were inserted into the pETDuet-1 vector between the NcoI/ScaI (MCS1) and EcoRV/XhoI (MCS2) restriction sites, respectively.

The plasmids KIF1A(1-393)-GCN4-3xmCit or KIF5C(1-560)-mCit were as previously used and were gifts by Dr Kristen Verhey (19, 83). The GFP-expressing pSUPER vectors contained the following shRNA sequences: Sept5 (5ʹ-GCGGTGAACAACTCTGAATGT-3ʹ), Sept6 (5′-GCCCATCGTGGAATTCATTGA-3′), or *Photinus pyralis* luciferase (control; 5ʹ-CGCTGAGTACTTCGAAATGTC-3ʹ) and were gifts of Dr Matthew Rasband (61). The plasmid pET28a-Strep-sfGFP-BICD2N was the gift of Dr Richard McKenney (84).

### Septin expression and purification

Recombinant septin complexes were prepared as described before (50, 85). The following cotransformations into *E. coli* BL21 (DE3) (Invitrogen) were performed: His-mCherry-SEPT2 and pnCS SEPT6/7-Strep(+1-57 bp SEPT7 N-term) (SEPT2/6/7), His-mCherry-SEPT5 and SEPT11/7-strep (SEPT5/7/11), and pnEA-vH_His-TEV-SEPT2-mCherry_SEPT6 and pnCS_SEPT7_SEPT9_i1-TEV-Strep (SEPT2/6/7/9). For the preparation of SEPT5/7/11, bacterial cultures were grown to A600 of 2 to 3 at 37 °C and induced with 1 mM IPTG for 4 h at 37 °C. Preparation of SEPT2/6/7 and SEPT2/6/7/9 required growth of bacterial cultures to A600 of 0.6 to 0.8 and induction with 0.5 mM IPTG for 16 h at 18 °C. After induction, cultures were kept on ice and centrifuged at 5000*g* for 10 min at 4 °C. For all three complexes, lysis buffer containing 300 mM KCl, 50 mM Tris–HCl, pH 8.0, 5 mM MgCl2, 0.25 mg/ml lysozyme, 10 mg/ml DNAse, 1 mM PMSF, and Bacterial Protease Arrest cocktail (G-Biosciences; 786–330) was used to lyse pelleted bacteria. Cell lysis was followed by sonication performed on ice (8 rounds of pulsing for 30 s followed by a 30 s pause between each round). Following sonication, lysates were centrifuged at 4 °C for 30 min at 20,000*g* and then filtered using 0.45 μm pore filter. Nickel-nitrilotriacetic acid agarose beads (0.8-1 ml) (745400.25; Macherey-Nagel) were loaded into gravity flow columns and equilibrated with buffer that contained 300 mM KCl, 50 mM Tris–HCl, pH 8.0, 5 mM MgCl2, and 10 mM imidazole. After column equilibration, the supernatant of each pellet was incubated with nickel-nitrilotriacetic acid agarose beads for 1 h and then allowed to flow through the column. Columns were then washed in equilibration buffer, and septin complexes were released from the column with elution buffer (300 mM KCl, 50 mM Tris–HCl, pH 8.0, 5 mM MgCl2, and 250 mM imidazole). The entire elution volume from the nickel-nitrilotriacetic acid column was loaded onto a StrepTactin Sepharose resin column (cat # 2-1201-002; IBA Lifescience) (GE Healthcare), which was equilibrated with 300 mM KCl, 5 mM MgCl2, and 50 mM Tris–HCl, pH 8.0. A final elution step was performed in 300 mM KCl, 5 mM MgCl2, 50 mM Tris–HCl, pH 8.0, and 2.5 mM d-Desthiobiotin (Sigma; D1411), which was followed by overnight dialysis at 4 °C in 300 mM KCl, 5 mM MgCl2, and 50 mM Tris–HCl, pH 8.0. DTT was added at a final concentration of 1 to 3 mM, and small aliquots were flash frozen for long term storage at −80 °C.

### Kinesin and DDB motors

COS-7 cells were transfected with truncated constitutively active motor domain constructs KIF1A(1-393)-GCN4-3xmCit or KIF5C(1-560)-mCit using Lipofectamine 2000 (Thermo Fisher Scientific). After 24 to 48 h, media was removed, cells were gently washed with ice cold PBS or buffer A (30 mM Hepes pH 7.4, 50 mM K-acetate pH 7.4, 2 mM Mg-acetate, 1 mM EGTA pH 7.4, 10% glycerol), and incubated for 20 min in lysis buffer (buffer A, 1 mM PMSF, 0.5% Triton X-100, EDTA-free protease inhibitor III (Calbiochem),1 mM ATP). Cell lysis was followed by centrifugation of extracts at 16,000*g* for 10 min. Additional ATP was added to extracts at a final concentration of 1 mM, and small aliquots were flash frozen for long term storage at −80 °C. DDB purification and motility assays were performed as previously described with the exception that endogenous dynein–dynactin adapter complexes were isolated from HEK293T cells rather than RPE-1 (84). In brief, bacteria were transformed with the plasmid pET28a-Strep-sfGFP-BICD2N, and the purified sfGFP-BICD2N was mixed with HEK293 lysates and Strep-Tactin beads overnight at 4 °C. Beads were washed and eluted with 3 mM desthiobiotin and 0.5 mM ATP.

### Microtubule coating with recombinant septin complexes and immunolabeling of microtubule-bound septins

Acid-washed glass coverslips (0.15 mm thick) were mounted on a glass slide using double sided tape to create 8 to 10 μl motility chambers. Taxol-stabilized microtubules were prepared by incubating unlabeled (80%), HiLyte647 (10%), and biotin conjugated (10%) porcine brain tubulin (Cytoskeleton Inc) in BRB80 (80 mM Pipes pH 6.9, 1 mM EGTA pH 6.9, 2 mM MgCl2, 10% glycerol) supplemented with 1 mM GTP at 37 °C for 35 min. The concentrated microtubule mix was incubated at 37 °C for 20 min following the addition of 10 μM taxol and then kept at room temperature while protected from light. To adhere biotinylated microtubules to the glass, each chamber was incubated with 5 mg/ml biotinylated bovine serum albumin (BSA) (Sigma) followed by 0.5 mg/ml neutravidin (Thermo Fisher Scientific) which was diluted in cold PBS. Chambers were incubated with taxol-stabilized microtubules diluted in BRB80 containing 1 mM GTP for 15 min, followed by blocking buffer (BRB80, 1 mg/ml BSA, 1% w/v Pluronic F-127 (Sigma), 10 μM taxol) for an additional 5 min. Chambers were then washed with 80 to 100 μl Hepes buffer (30 mM Hepes–KOH pH 7.4, 50 mM of KOAc pH 7.4, 2 mM of MgOAc, 1 mM of EGTA–KOH pH 7.4, and 10% glycerol). Septin complexes were diluted in the Hepes buffer supplemented with 0.1% w/v Pluronic F-127, 0.1 mg/ml BSA, and 10 μM Taxol (Hepes dilution buffer) before applying to the chambers and incubated for 15 min at room temperature. A final wash with 80 to 100 μl Hepes dilution buffer was done before imaging with TIRF microscopy using the DeltaVision OMX V4 imaging platform (GE Healthcare) with 60X/1.49 NA objective (Olympus), sCMSO pco.edge cameras.

After microtubule coating with septin complexes, microtubule-bound septins were immunolabeled by first blocking for 10 min at room temperature with Hepes buffer supplemented with 1% w/v Pluronic F-127, 2% BSA, and 10 μM taxol. Prior to labeling, rabbit anti-SEPT6 (S6CU; a gift from Dr Makoto Kinoshita, Nagoya University), rabbit anti-SEPT7 (IBL America 18991), rabbit anti-SEPT9 (ProteinTech 10769-I-AP), or rabbit anti-SEPT11 (MilliporeSigma ABN1342) antibodies were diluted in Hepes buffer and conjugated with 5 μl of the Zenon Alexa Fluor 488 rabbit IgG labeling reagent for 5 min at room temperature. The Zenon blocking reagent (rabbit IgG; 5 μl) was then added to the mix and incubated for 5 min at room temperature before further dilution in 30 μl of Hepes buffer. The diluted mix was applied to the chamber and incubated for 15 min at room temperature before a final wash with 80 to 100 μl of Hepes buffer. Chambers were imaged with TIRF microscopy using the DeltaVision OMX V4 imaging platform (GE Healthcare) with 60X/1.49 NA objective (Olympus), sCMSO pco.edge cameras, and the softWoRx software (https://download.cytivalifesciences.com/cellanalysis/download_data/softWoRx/7.0.0/SoftWoRx.htm).

### Single molecule motility assays

Stabilized microtubules were made by incubating unlabeled (80%), HiLyte647 (10%), and biotin-conjugated (10%) porcine brain tubulin (Cytoskeleton Inc) in BRB80 (80 mM Pipes pH 6.9, 1 mM EGTA pH 6.9, 2 mM MgCl2, 10% glycerol) supplemented with 1 mM GTP at 37 °C for 0.5 to 1 h. The concentrated microtubule mix was incubated at 37 °C for 15 to 30 min following the addition of Taxol (10 μM) and then kept at room temperature while protected from light. Acid-washed glass coverslips (0.15 mm thick) were mounted on a glass slide using double sided tape in order to create 8 to 10 μl motility chambers. To adhere biotinylated microtubules to the glass, each chamber was incubated with 5 mg/ml biotinylated BSA (Sigma) followed by 0.5 mg/ml Neutravidin (Thermo Fisher Scientific) which was diluted in cold PBS. Chambers were incubated with taxol-stabilized microtubules diluted in BRB80 containing 1 mM GTP for 10 min, followed by blocking buffer (BRB80, 1 mg/ml BSA, 1% w/v Pluronic F-127 (Sigma), 10 μM taxol) for an additional 10 min.

Extracts containing kinesin motors were diluted in buffer A supplemented with (0.1 mg/ml BSA, 0.1% Pluronic F-127, 2 mM ATP, 10 μM taxol) and a glucose-based oxygen scavenging system (0.035 mg/ml catalase, 4.5 mg/ml D-glucose, 0.2 mg/ml glucose oxidase, 30 mM b-mercaptoethanol in buffer A) in the final motility mix. For motility assays performed in the presence of septin complexes, blocked microtubules were incubated with mCherry-SEPT2/6/7, mCherry-SEPT5/11/7, or mCherry-SEPT2/6/7/9 diluted in buffer A supplemented with 0.1 mg/ml BSA, 0.1% Pluronic F-127, and 10 μM taxol for 10 min. Chambers were washed with buffer A prior to the addition of the final motility mix containing kinesin motors. The motility of microtubule motors was recorded using time-lapse TIRF microscopy and was performed at room temperature (kinesins acquired at five frames/s for 2 min, DDB acquired at one frame/s for 2 min).

### Neuronal culture and transfection

Embryonic (E18) rat hippocampal neurons were isolated and cultured as previously described (53). DIV10 neurons were transfected with Lipofectamine 3000 (Thermo Fisher Scientific) and fixed after 96 h. Depletion of Septin 5, Septin 6, or *P. pyralis* luciferase was performed with GFP-expressing pSUPER vectors, a gift of Dr Matthew Rasband (61), and contained the following shRNA sequences: Sept5 (5ʹ-GCGGTGAACAACTCTGAATGT-3ʹ), Sept6 (5′-GCCCATCGTGGAATTCATTGA-3′), or *P. pyralis* luciferase (control; 5ʹ-CGCTGAG- TACTTCGAAATGTC-3ʹ). An E.Z.N.A. Endo-Free Plasmid DNA Midi Kit (Omega Bio-Tek, D6915-03) was used to prepare all pSUPER plasmids.

### Immunofluorescence

Cultured DIV10-DIV14 neurons were fixed with warm fixation buffer containing PBS supplemented with 4% sucrose and 3% paraformaldehyde for 10 min, permeabilized with 0.1% Triton X-100 in GDB (0.2% gelatin, 450 mM NaCl, 30 mM sodium phosphate pH 7.4) for 10 min, and blocked with GDB for an additional 30 min (Fig. 6). Primary and secondary antibodies were diluted in GDB and centrifuged at 100,000*g* at 4 °C for 10 min before adding onto cells. Following a blocking step, primary antibodies were incubated overnight at 4 °C. The following day, cells were washed with GDB and secondary antibodies diluted in GDB were incubated at room temperature for 1 to 1.5 h. Neurons transfected with pSUPER plasmids were also incubated with 1 mg/ml DAPI (Sigma) for 10 min prior to mounting. FluorSave mounting medium (EMD Millipore) was used to preserve fixed samples, which were allowed to dry for 1 to 2 h before imaging or storage at 4 °C overnight.

To stain for microtubule-associated septins, neurons were incubated in warm pre-extraction buffer (80 mM Pipes pH 6.9, 1 mM EGTA, 150 mM NaCl, 7 mM MgCl2, 5 mM D-glucose, 0.25% glutaraldehyde, and 0.05% Triton X-100) for 20 s, immediately rinsed in warm pre-conditioned neurobasal media, and fixed with warm fixation buffer (PBS, 4% paraformaldehyde, 4% sucrose) for 10 min (Figs. 5 and S4). Following fixation, samples were quenched with 75 mM NH4Cl in PBS, permeabilized with GDB containing 0.05% Triton X-100 for 5 min, and blocked with GDB for 30 min prior to performing the staining procedure described above.

### Antibodies

DIV10-DIV14 hippocampal neurons were stained with chicken anti-MAP2 (1:1000; EMD Millipore; AB5543), mouse anti-GM130 (1:200, BD Transduction; 610823), rabbit anti-GOLGA2/GM130 (1:500; Proteintech; 11308-1-AP; Fig. S5), mouse anti-dynein (1:500; EMD Millipore; MAB1618), rabbit anti-SEPT5 (1:500, Santa Cruz; sc-20040), rabbit anti-SEPT6 (1:200; gift from Dr Makoto Kinoshita, Nagoya University), and mouse mAb anti-SEPT6 (clone 9E7; gift from Dr Ian Macara) (86). Donkey anti-rabbit, anti-chicken, and anti-mouse secondary antibodies conjugated with aminomethylcoumarin acetate, Alexa488, or Alexa647 (Jackson ImmunoResearch Laboratories) were used at 1:200.

### Microscopy

All TIRF microscopy (*in vitro* motility assays, Fig. 5, *A*–*D*) was performed with the TIRF module of a Deltavision OMX V4 inverted microscope using a 60X/1.49 NA oil immersion objective and cCMOS pco.edge cameras (PCO) and the softWoRx software. *In vitro* motility assays were performed at room temperature. In Figure 5, *E* and *F*, images DIV10 neurons were acquired on a Leica THUNDER imager with an inverted DMI8 microscope stand, HC PL APO 63x/1.40 oil objective, and a Leica-K8-A21M726013 camera. Images were processed with the Large Volume Computational Clearing module of the Leica LAS X software (https://www.leica-microsystems.com/products/microscope-software/p/leica-las-x-ls/). In Figure 5*G*, images were acquired with the Leica Stellaris 5 confocal using a 63X/1.4 NA oil immersion objective, zoom of 1X-3X, and 0.2 μm step size for z-stack collection. Images were processed with LIGHTNING convolution module for enhanced lateral resolution. In Figures 6 and 3*D*, images of DIV14 neurons were acquired on a Zeiss AxioObserver Z1 inverted microscope equipped with a 63x/1.4 NA oil immersion objective and a Hamamatsu Orca-R2 CCD camera.

### Quantification of microtubule-bound septin complexes

Quantification of the mCherry fluorescence of recombinant septin complexes was performed in Fiji/ImageJ using the plot profile function. A straight line with thickness of 1 was drawn along individual microtubules with >10 μm length. For fluorescence background subtraction, a 10 μm-long straight was drawn at a distance of at least ∼2 μm away from any microtubule. The mean fluorescence intensity of each microtubule was plotted after subtracting the mean fluorescence intensity of the background. All statistical analyses were performed in GraphPad Prism software 9 (https://www.graphpad.com/) and Microsoft Excel to calculate mean values, S.E.M., and SD, and statistical significance (*p* values) was derived using a Welch's one way ANOVA test with post hoc Dunnett's T-3 test for multiple pairwise comparisons.

### Analysis of *in vitro* motility

Individual motors were tracked manually using frame-by-frame analysis in ImageJ/Fiji. The movement path of each motor (start and end frame) was traced using the segmented line tool, saved as a region of interest, and used to determine motor run lengths. Only kinesin motors that moved processively for at least 0.6 s (3 frames) and DDB motors that were processive for at least 3 s were tracked. For both kinesin and DDB, only microtubules of 10 μm length or longer were considered for analysis and velocities were determined by dividing the run length by total event duration. Landing rates, pausing, and immotile events were determined by generating kymographs created using a region of interest along the length of each microtubule. Landing events were defined as events of motor landing onto the microtubule lattice followed by sustained unidirectional movement that lasted for more than three consecutive frames without reversal of direction or diffusive movement and resolved by a diagonal line in kymographs of movement. The number of landing events were scored for individual microtubules and divided by the length of the microtubule and the duration of the video to extract the landing rates. Pause events were defined as processive events which contained at least a single pause for three frames (600 ms) or longer. The percentage of pausing events was calculated by dividing the total number of pausing events by the total number of their respective processive events per microtubule and multiplying it by 100. Immotile events were defined as particles that bound to the microtubule for at least three frames (600 ms) or more and did not move processively at all. The frequency of immotile events was calculated by dividing the number of immotile events by the length of the microtubule and the duration of the time-lapse capture.

### Golgi polarity and morphology analysis

DIV14 neurons transfected with pSUPER plasmids (GFP) were stained with antibodies to GM130, which labeled the Golgi complex, MAP2 to mark neuronal dendrites, and DAPI. Cells with condensed DAPI-labeled chromosomes and other brightly labeled nuclear abnormalities, which are indicative of apoptosis, were excluded from analysis. Golgi complexes (GM130) that were positioned at the base of a single MAP2-labeled dendrite were scored as polarized, and Golgi complexes which span the entry points of multiple dendrites were scored as nonpolarized. Golgi complexes that were either scattered throughout the cell body or condensed near the nucleus were also scored as nonpolarized. Morphology of the Golgi complex was classified as tubulated/ribbon if it consisted of elongated ribbons, clustered/condensed if Golgi ribbons or stacks were tightly coalesced into a kidney-shaped bolus, and fragmented if consisted of numerous smaller stacks that were disconnected and dispersed throughout the cell body and dendrites. The Golgi was categorized as deployed if tubular ribbons were extended at least five microns into the shaft of one or more dendrites. Within each classification (polarity, morphology, deployment), the percentage of cells was determined by dividing the n in each category by the total number of cells scored and multiplying by 100.

### Quantification of Golgi (GM130) colocalization with SEPT5

Three-dimensional z-series stacks of confocal microscopy images of DIV10 hippocampal neurons were imported into the IMARIS (9.9.1) analysis software (https://imaris.oxinst.com/). Background subtraction was performed on both the GM130 and SEPT5 channels with a filter width of 1 μm. A 3D mask was generated for each channel by thresholding fluorescence signals to a range that best fit the raw signal without including background fluorescence. Prior to fluorescence segmentation, the approximate center z-plane was determined visually by selecting the plane where the majority of the GM130 signal was visible. The colocalization tool was then used to generate a channel representing the 3D overlap between the GM130 and SEPT5 channels. Using this channel, the IMARIS software automatically generated the percentage volume overlap and Mander's coefficients between the GM130 and SEPT5 channels.

### Quantification of dynein levels on Golgi (GM130) stacks

Quantifications were performed on the midplane optical sections of three-dimensional z-series stacks, which were collected with the Zeiss AxioObserver microscope and Slidebook 6.0 software (https://www.intelligent-imaging.com/slidebook). The center of the z-plane was determined visually by selecting the plane where the majority of the GM130 signal was visible. Fluorescence thresholding was used to create a mask around the GM130 and dynein signals after applying no-neighbors deconvolution to improve signal-to-noise ratio. Due to background staining of the nucleus with the rabbit GM130 antibody, the nuclear area was excluded from quantification by generating a second mask that overlapped with the nuclear area. Using the mask operations tool, the nuclear mask was subtracted from the Golgi mask to generate the Golgi area where dynein fluorescence signal was quantified. The total surface area of the masked Golgi (GM130) ribbons and stacks and the sum fluorescence intensity of dynein were derived with the mask statistics tool of the Slidebook 6.0 software.

### Statistical analysis

The GraphPad Prism 8 or Microsoft Excel software was used to calculate mean values, SEM, SD, and to perform statistical tests for deriving *p* values. Data were first analyzed with the D’Agostino and Kolmogorov–Smirnov tests to test for normal distribution. For pairwise comparisons of data with normal distributions, a student’s *t* test was used if SDs were equal or a Welch's *t* test if SDs were unequal. The Mann–Whitney test was used for non-normally distributed data. For statistical analysis of multiple groups with normally distributed data, a one-way ANOVA was performed with a post hoc Dunnett test for multiple pairwise comparisons. For data that were not normally distributed, a nonparametric Kruskal–Wallis ANOVA test was performed with a post hoc Dunn's test for pairwise comparisons. In Figure 6, categorical data were statistically analyzed with the chi square test. Statistical significance (*p* values) of pairwise comparisons was derived in GraphPad Prism, while statistical significance for the entire group cohort was derived using an automated on-line chi square calculator (https://www.socscistatistics.com/tests/chisquare2/default2.aspx) and results were reported in the format: *Χ* ^2^ (degrees of freedom, *N* = sample size) = chi square statistic value, *p* = *p* value. Results with *p* values of < 0.05 were considered to be statistically significant. Data with *p* values < 0.05 were determined as statistically significant.

## Data availability

All data described are provided and included in the figures of this manuscript.

## Supporting information

This article contains supporting information.

## Conflict of interest

The authors declare that they have no conflicts of interest with the contents of this article.
