# Supplementary material for: Microtubule-associated septin complexes modulate kinesin and dynein motility with differential specificities
Source: J Biol Chem. 2023 Jul 24;299(9):105084. doi: 10.1016/j.jbc.2023.105084 (PMC10463263; doi:10.1016/j.jbc.2023.105084)
Supplement: Supporting Figures S1–S5 [file mmc1.pdf]

Supplementary Information for

Microtubule-associated septin complexes modulate kinesin and dynein motility with differential specificities

Yani Suber[1], Md Noor A Alam[1], Konstantinos Nakos[1,2,3], Priyanka Bhakt[1,4] and Elias T Spiliotis[1,*]

[1]Department of Biology, Drexel University, Philadelphia, PA 19104

[2]Present address: Department of Molecular Biology, Massachusetts General Hospital, Boston, MA 02114

[3]Present address: Department of Genetics, Harvard Medical School, Boston, MA 02115

[4]Present address: Department of Pathology and Laboratory Medicine, University of Pennsylvania Perelman School of Medicine, Philadelphia, PA 19104

[*]Correspondence: Elias Spiliotis, ets33@drexel.edu

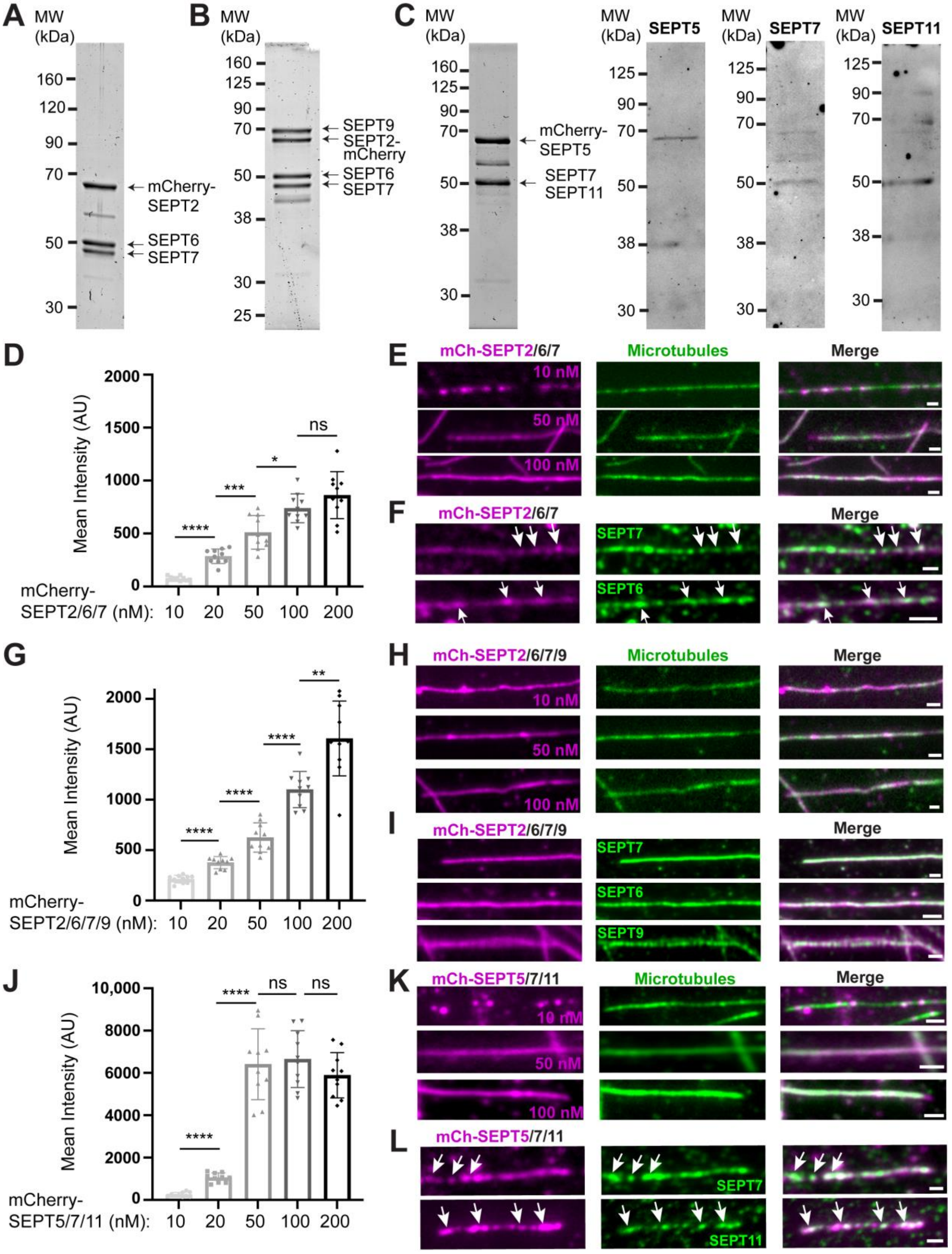
A
MW (kDa)
160
120
90
70
50
30
mCherry-SEPT2
SEPT6
SEPT7
B
MW (kDa)
160
125
90
70
50
38
30
25
SEPT9
SEPT2-mCherry
SEPT6
SEPT7
C
MW (kDa)
160
125
90
70
50
38
30
mCherry-SEPT5
SEPT7
SEPT11
SEPT5
SEPT7
SEPT11
D
Mean Intensity (AU)
mCherry-SEPT2/6/7 (nM): 10 20 50 100 200
ns
E
mCh-SEPT2/6/7
Microtubules
Merge
10 nM
50 nM
100 nM
F
SEPT7
SEPT6
G
mCherry-SEPT2/6/7/9 (nM): 10 20 50 100 200
H
mCh-SEPT2/6/7/9
I
SEPT9
J
10,000
mCherry-SEPT5/7/11 (nM): 10 20 50 100 200
K
mCh-SEPT5/7/11
L

Figure S1. Decoration of microtubules with purified mCherry-tagged SEPT2/6/7, SEPT2/6/7/9 and SEPT5/7/11 complexes.

(A-B) Coomassie blue-stained SDS-PAGE gels show the purified mCherry-tagged SEPT2/6/7 and SEPT2/6/7/9 complexes and their subunits, which were used to coat microtubules for in vitro motility assays.

(C) Coomassie blue-stained SDS-PAGE gel (far left) shows the purified mCherry-tagged SEPT5/7/11 complex, and western blots of the purified mCherry-SEPT5/7/11 complex show the mCherry-His-SEPT5, SEPT7 and SEPT11 subunits after probing with antibodies to His, SEPT7 and SEPT11, respectively. Note that SEPT7 and SEPT11 have overlapping molecular weights and appear as a single band in the Coomassie-stained gel.

(D) Mean (± S.D.) fluorescence intensity of microtubule-bound mCherry-SEPT2/6/7 following incubation of microtubules (n = 10) with mCherry-SEPT2/6/7 at concentrations of 10 nM (74 ± 20 A.U.), 20 nM (286 ± 68 A.U.), 50 nM (511 ± 159 A.U.), 100 nM (739 ± 136 A.U.) and 200 nM (863 ± 222 A.U.). Data were statistically analyzed with a Welch's one way ANOVA test with post-hoc Dunnett's T3 test for multiple pair-wise comparisons; ns, not significant ($p > 0.05$); *, $p = 0.01$; **, $p = 0.006$; ****, $p < 0.0001$

(E) TIRF microscopy images show representative examples of microtubule-bound mCherry-SEPT2/6/7 after coating with 10 nM, 50 nM and 100 nM of mCherry-SEPT2/6/7. Scale bars, 1 μm.

(F) TIRF microscopy images show immunofluorescence staining of mCherry-SEPT2/6/7-bound microtubules with AlexaFluor488-conjugated antibodies against SEPT7 and SEPT6. Microtubules were coated with 100 nM mCherry-SEPT2/6/7 prior to staining. Discontinuous coating of microtubules with mCherry-SEPT2/6/7 is due to partial dissociation of septin complexes during the blocking and staining steps of the immunofluorescence protocol. Arrows point to discrete microtubule domains of mCherry-SEPT2/6/7 enrichment, which were also labeled with SEPT7 and SEPT6 antibodies. Scale bars, 1 μm.

(G) Mean (± S.D.) fluorescence intensity of microtubule-bound mCherry-SEPT2/6/7/9 following incubation of microtubules (n = 10) with mCherry-SEPT2/6/7/9 at concentrations of 10 nM (213 ± 38 A.U.), 20 nM (377 ± 60 A.U.), 50 nM (626 ± 145 A.U.), 100 nM (1,101 ± 179 A.U.) and 200 nM (1,608 ± 371 A.U.). Data were statistically analyzed with a Welch's one way ANOVA test with post-hoc Dunnett's T3 test for multiple pair-wise comparisons. **, p = 0.001-0.007; ****, $p < 0.0001$

(H) TIRF microscopy images show representative examples of microtubule-bound mCherry-SEPT2/6/7/9 after coating with 10 nM, 50 nM and 100 nM of mCherry-SEPT2/6/7/9. Scale bars, 1 μm.

(I) TIRF microscopy images show immunofluorescence staining of mCherry-SEPT2/6/7/9-bound microtubules with AlexaFluor488-conjugated antibodies against SEPT7, SEPT6 and SEPT9. Microtubules were coated with 100 nM mCherry-SEPT2/6/7/9 prior to staining. Scale bars, 1 μm.

(J) Mean (± S.D.) fluorescence intensity of microtubule-bound mCherry-SEPT5/7/11 following incubation of microtubules (n = 10) with mCherry-SEPT5/7/11 at concentrations of 10 nM (254 ± 103 A.U.), 20 nM (1,063 ± 221 A.U.), 50 nM (6,413 ± 1,672 A.U.), 100 nM (6,653 ± 1,341 A.U.) and 200 nM (5,893 ± 1,070 A.U.). Quantification was performed on images acquired with higher laser power than in the coating experiments with mCherry-SEPT2/6/7 (D) and mCherry-SEPT2/6/7/9 (G). Data were statistically analyzed with a Welch's one way ANOVA test with post-hoc Dunnett's T3 test for multiple pair-wise comparisons; ns, not significant ($p > 0.05$); ****, $p < 0.0001$

(K) TIRF microscopy images show representative examples of microtubule-bound mCherry-SEPT5/7/11 after coating with 10 nM, 50 nM and 100 nM of mCherry-SEPT5/7/11. Scale bars, 1 μm.

(L) TIRF microscopy images show immunofluorescence staining of mCherry-SEPT5/7/11-bound microtubules with AlexaFluor488-conjugated antibodies against SEPT7 and SEPT11. Microtubules were coated with 100 nM mCherry-SEPT5/7/11 prior to staining. Discontinuous coating of microtubules with mCherry-SEPT5/7/11 is due to partial dissociation of septin complexes during the blocking and staining steps of the immunofluorescence protocol. Arrows point to discrete microtubule domains of mCherry-SEPT5/7/11 enrichment, which were also labeled with SEPT7 and SEPT11 antibodies. Scale bars, 1 μm.

Data were statistically analyzed with a Welch's one way ANOVA test with post-hoc Dunnett's T3 test for multiple pair-wise comparisons.

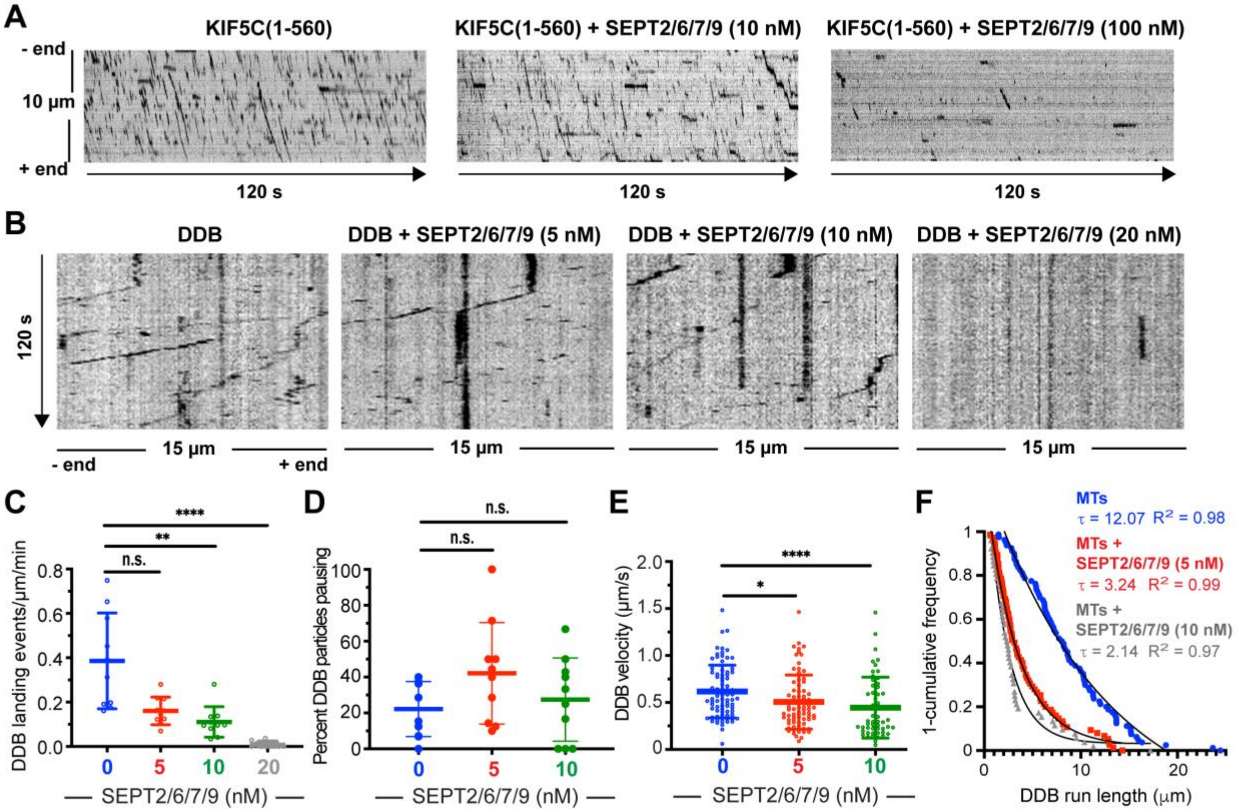

Figure S2. Microtubule-associated SEPT2/6/7/9 complexes inhibit the motility of KIF5C and DDB

(A) Representative kymographs of KIF5C(1-560)-mCit motility on uncoated microtubules (left), and microtubules coated with 10 nM (middle) and 100 nM (right) of mCherry-SEPT2/6/7/9. Motile particles appear as diagonal lines and static particles appear as horizontal lines, respectively. Time is in the x-axis and distance in the y-axis, which indicates the relative orientation of microtubule plus and minus ends.

(B) Representative kymographs of DDB-GFP motility on uncoated microtubules (far left) and microtubules coated with 5 nM, 10 nM and 20 nM DDB-GFP. Motile particles appear as diagonal lines and static particles appear as vertical lines, respectively. Distance is in the x-axis, which indicates the relative orientation of microtubule plus and minus ends, and time is in the y-axis.

(C) Mean (± S.D.) landing rates of DDB particles on uncoated microtubules (0.39 ± 0.22 events/μm/minute; *n* = 10 microtubules) and microtubules (n = 10), which were coated with 5 nM (0.16 ± 0.06 events/μm/minute), 10 nM (0.11 ± 0.07 events/μm/minute) and 20 nM (0.01 ± 0.01 events/μm/minute) mCherry-SEPT2/6/7/9. n.s., not significant ($p > 0.05$); **, $p = 0.009$; ****, $p < 0.0001$

(D) Mean (± S.D.) percentage of DDB-GFP particles that pause on uncoated microtubules (22.19% ± 15.35%; $n = 10$) and microtubules ($n = 10$) which were coated with 5 nM (42.12% ± 28.30%) and 10 nM mCherry-SEPT2/6/7/9 (27.45% ± 23.23%; $n = 10$). n.s., not significant

(E) Mean (± S.D.) velocity of DDB particles on uncoated microtubules (0.62 ± 0.28 μm/s; $n = 80$) and microtubules, which were coated with 5 nM (0.51 ± 0.29 μm/s; $n = 75$) and 10 nM mCherry-SEPT2/6/7/9 (0.45 ± 0.32 μm/s; $n = 52$). *, $p = 0.016$; ****, $p < 0.0001$

(F) One-cumulative distribution plot of the run lengths of DDB-GFP particles on uncoated microtubules ($n = 80$) and microtubules coated with 10 nM ($n = 75$) or 100 nM ($n = 52$) mCherry-SEPT2/6/7/9. Data were fit to one-phase exponential decay with a decay constant τ (run length), which is shown with the R^2 fit value. The mean (± S.D.) run length value on uncoated microtubules was 7.89 ± 3.88 μm ($n = 80$), and the mean (± S.D.) run length values on microtubules with 10 nM and 100 nM mCherry-SEPT2/6/7/9 were 4.4 ± 3.5 μm ($n = 75$) and 3.4 ± 3.27 μm ($n = 52$), respectively. Reduction of run length values for 10 nM and 100 nM mCherry-SEPT2/6/7/9 in comparison to uncoated microtubules was statistically significant ($p < 0.0001$).

Data were statistically analyzed with one-way ANOVA and a post-hoc Dunnett test for multiple comparisons (B-C) or Kruskal-Wallis test with post-hoc Dunn's test for multiple pairwise comparisons (D-E).

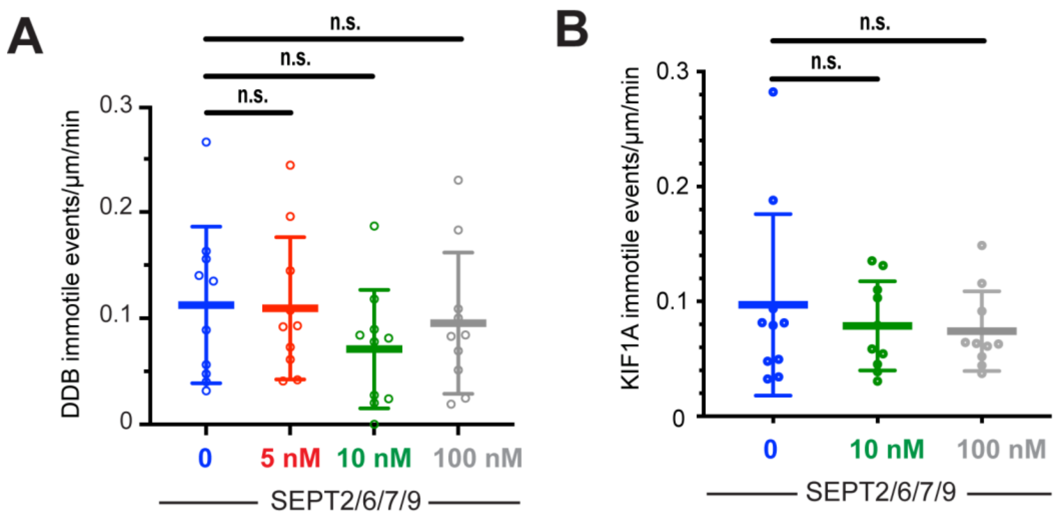

Figure S3. Microtubule-associated SEPT2/6/7/9 complexes have no impact on the immotile fraction of DDB and KIF1A.

(A) Mean number (± S.D.) of immotile DDB particles per micrometer of uncoated microtubules (0.11 ± 0.07 particles/µm; n = 10 microtubules) and microtubules (n = 10) coated with 5 nM (0.11 ± 0.07 particles/µm), 10 nM (0.07 ± 0.06 particles/µm) and 100 nM mCherry-SEPT2/6/7/9 (0.10 ± 0.07 particles/µm) per minute of imaging. n.s., not significant ($p > 0.05$)

(B) Mean number (± S.D.) of immotile KIF1A(1-393)GCN4-3XmCit particles per micrometer of uncoated microtubules (0.10 ± 0.08 particles/µm; n = 10 microtubules) and microtubules (n = 10) coated with 10 nM (0.08 ± 0.04 particles/µm) and 100 nM (0.07 ± 0.03 particles/µm) mCherry-SEPT2/6/7/9 per minute of imaging. n.s., not significant ($p > 0.05$)

Data were statistically analyzed with one-way ANOVA and a post-hoc Dunnett test for multiple comparisons (A) or Kruskal-Wallis test with post-hoc Dunn's test for multiple pairwise comparisons (B). n.s., not significant ($p > 0.05$).

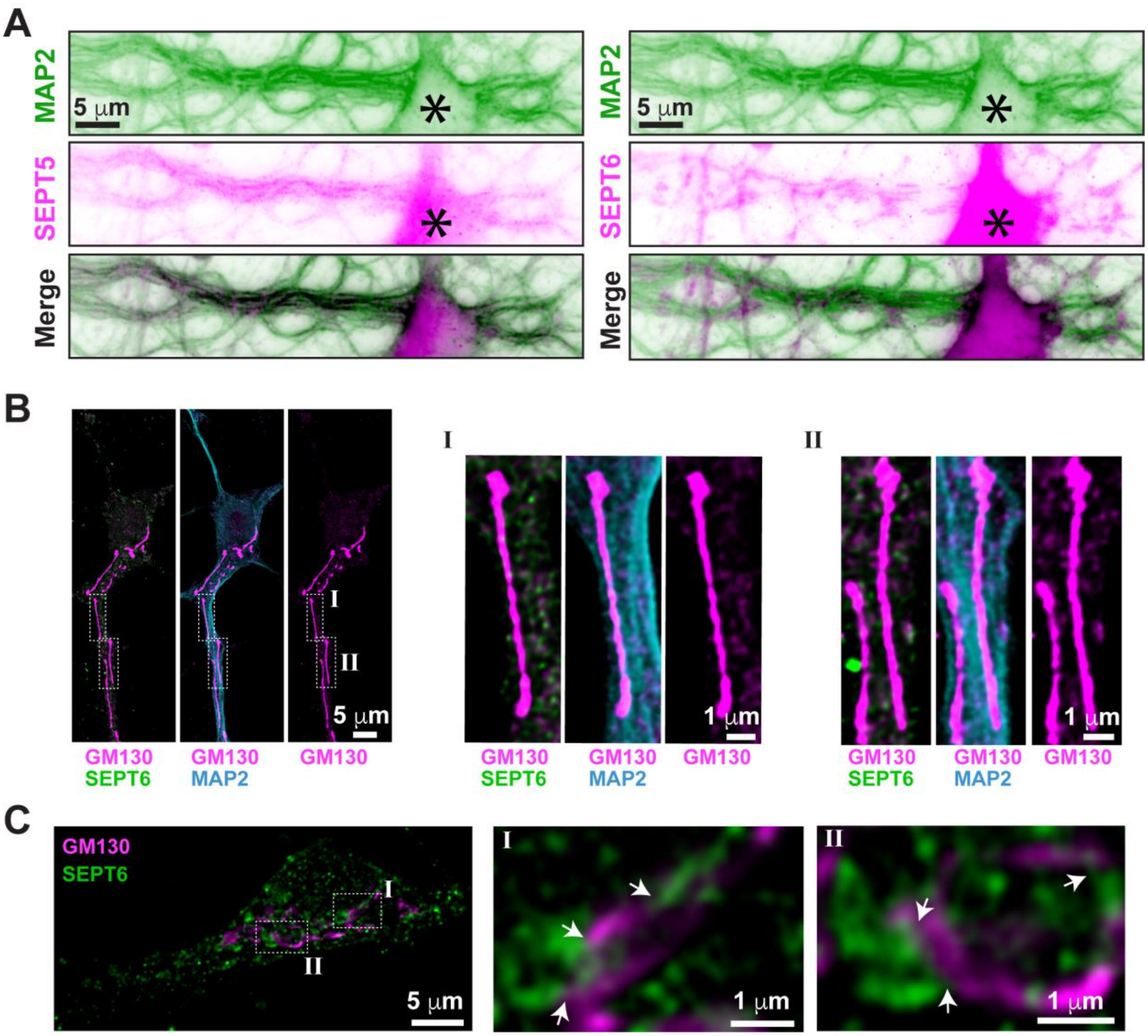

Figure S4. SEPT6 does not colocalize with SEPT5 and Golgi in rat hippocampal neurons.

(A) TIRF microscopy images of a primary rat hippocampal neuron (DIV14), which was stained with antibodies to SEPT5 (inverted forest green), SEPT6 (inverted forest green) and MAP2 (inverted magenta). SEPT5 colocalization with MAP2 appears in black color. Asterisks denote the cell body of the neuron. Scale bars, 5 μm.

(B) Super-resolution confocal microscopy of a primary embryonic rat hippocampal neuron (DIV10), which was stained for SEPT6 (green), MAP2 (cyan) and GM130 (magenta), and contains tubular Golgi membranes deployed into the principal dendrite. Regions outlined with dashed lines (1, 2) are shown in higher magnification. Scale bars, 5 μm and 1 μm (magnified regions).

(C) Deconvolution wide-field microscopy image of the cell body of a primary embryonic rat hippocampal neuron (DIV10) stained for GM130 and SEPT6. Regions outlined in dashed rectangles are shown in higher magnification. Arrows point to end-to-end (I) and orthogonal (II) contacts of GM130-labeled Golgi membranes with SEPT6 filaments. Scale bars, 5 μm and 1 μm (magnified regions I and II).

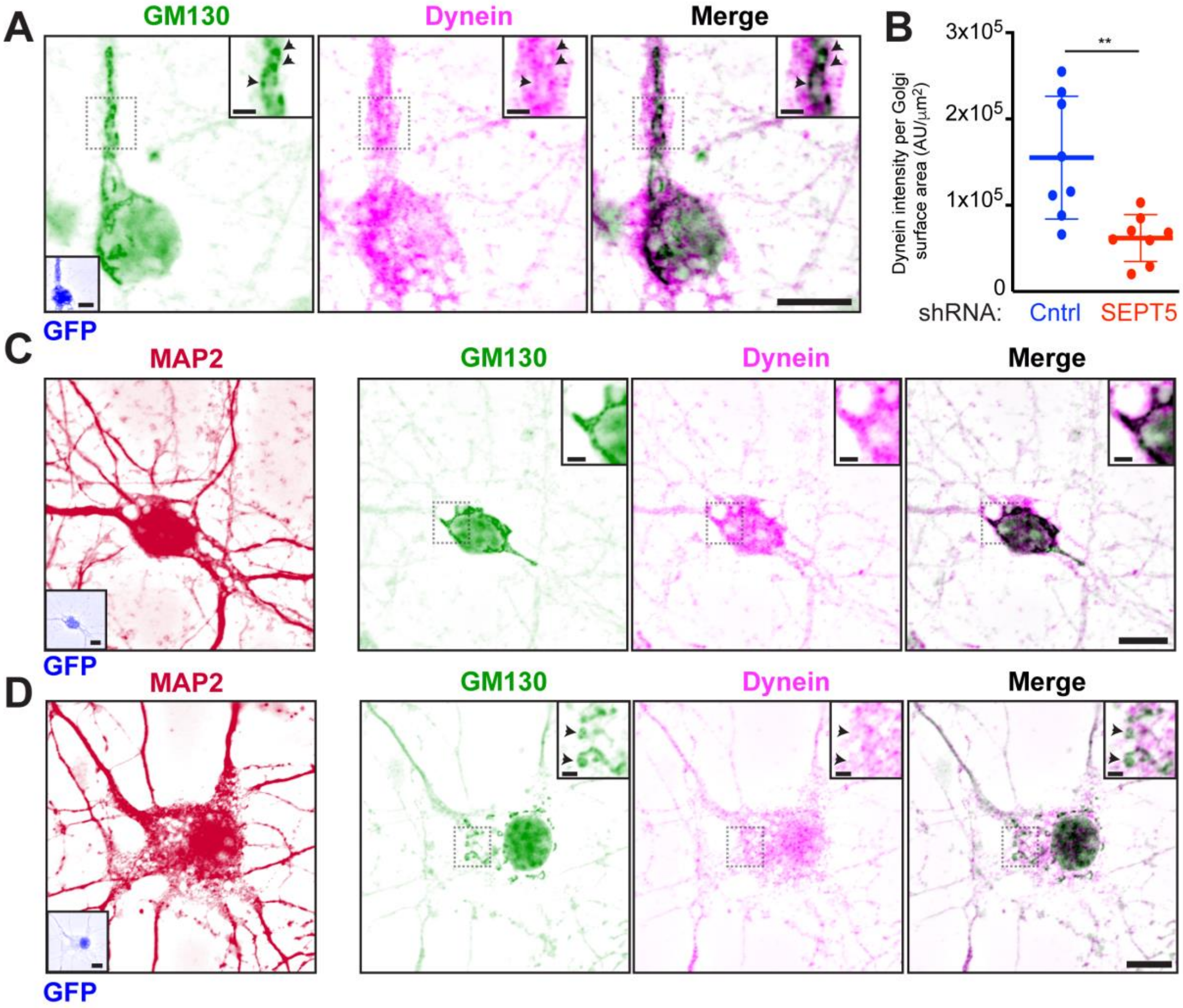

Figure S5. SEPT5 knock down reduces the levels of dynein at Golgi membranes.

(A) Images show a rat DIV13 hippocampal neuron, which expressed control non-targeting shRNA and GFP (lower left inset; inverted blue), and it was stained with antibodies against dynein (inverted magenta) and Golgi (GM130; inverted green). Images were obtained with wide-field fluorescence microscopy and processed with no-neighbors deconvolution. Arrowheads (upper right insets) point to dynein localization to Golgi ribbon domains and stacks. Scale bars, 10 μm and 2 μm (upper right insets).

(B) Mean (± S.D.) fluorescence intensity of dynein per surface area of Golgi (GM130) per cell; quantification was limited to all Golgi regions that did not overlap with the nucleus due to background staining of the latter with anti-GM130. Quantifications were performed in rat DIV13 hippocampal neurons (n = 8) after transfection with plasmids encoding GFP and control

(155,164 ± 71,150 A.U./μm^2) or SEPT5 shRNAs (61,957 ± 27,149 A.U./μm^2) at DIV10. Data were statistically analyzed with an unpaired t-test with Welch's correction. **, $p = 0.004$
(C-D) Images show rat DIV13 hippocampal neurons, which were transfected at DIV10 with plasmids expressing simultaneously GFP (inverted blue, lower left inset) and control non-targeting shRNA against luciferase (C) or shRNA against SEPT5 (D). Neurons were stained with antibodies against MAP2 (inverted bordeaux), dynein (inverted magenta) and Golgi (GM130; inverted green). Insets (upper right) show regions of Golgi membrane localization in higher magnification. Arrowheads point to lack of dynein enrichment at the Golgi stacks of a neuron transfected with SEPT5 shRNA. Images were acquired with wide-field fluorescence microscopy and processed with no neighbors deconvolution. Scale bars, 10 μm and 2 μm (upper right insets).
